# Bis(*N*,*N*-di­ethyl­dithio­carbamato-κ^2^ *S*,*S*′)(3-hy­droxy­pyridine-κ*N*)zinc and bis­[*N*-(2-hy­droxy­eth­yl)-*N*-methyldithio­carbamato-κ^2^ *S*,*S*′](3-hy­droxy­pyridine-κ*N*)zinc: crystal structures and Hirshfeld surface analysis

**DOI:** 10.1107/S205698901601728X

**Published:** 2016-11-01

**Authors:** Mukesh M. Jotani, Hadi D. Arman, Pavel Poplaukhin, Edward R. T. Tiekink

**Affiliations:** a Department of Physics, Bhavan’s Sheth R. A. College of Science, Ahmedabad, Gujarat 380 001, India; b Department of Chemistry, The University of Texas at San Antonio, One UTSA Circle, San Antonio, Texas 78249-0698, USA; c Chemical Abstracts Service, 2540 Olentangy River Rd, Columbus, Ohio, 43202, USA; d Research Centre for Crystalline Materials, Faculty of Science and Technology, Sunway University, 47500 Bandar Sunway, Selangor Darul Ehsan, Malaysia

**Keywords:** crystal structure, zinc, di­thio­carbamate, hy­droxy­pyridine, hydrogen bonding, Hirshfeld surface analysis

## Abstract

The common feature of the mol­ecular structures of the title compounds, [Zn(C_5_H_10_NS_2_)_2_(C_5_H_5_NO)], (I), and [Zn(C_4_H_8_NOS_2_)_2_(C_5_H_5_NO)], (II), are NS_4_ donor sets derived from *N*-bound hy­droxy­pyridyl ligands and asymmetrically chelating di­thio­carbamate ligands. The resulting coordination geometries are highly distorted, being inter­mediate between square pyramidal and trigonal bipyramidal for both independent mol­ecules comprising the asymmetric unit of (I), and significantly closer towards square pyramidal in (II). The key feature of the mol­ecular packing in (I) is the formation of centrosymmetric, dimeric aggregates sustained by pairs of hy­droxy-O—H⋯S(di­thio­carbamate) hydrogen bonds. The aggregates are connected into a three-dimensional architecture by methyl­ene-C—H⋯O(hy­droxy) and methyl-C—H⋯π(chelate) inter­actions. With greater hydrogen-bonding potential, supra­molecular chains along the *c* axis are formed in the crystal of (II), sustained by hy­droxy-O—H⋯O(hy­droxy) hydrogen bonds, with ethyl­hydroxy and pyridyl­hydroxy groups as the donors, along with ethyl­hydroxy-O—H⋯S(di­thio­carbamate) hydrogen bonds. Chains are connected into layers in the *ac* plane by methyl­ene-C—H⋯π(chelate) inter­actions and these stack along the *b* axis, with no directional inter­actions between them. An analysis of the Hirshfeld surfaces clearly distinguished the independent mol­ecules of (I) and reveals the importance of the C—H⋯π(chelate) inter­actions in the packing of both (I) and (II).

## Abstract 2

Highly-distorted five-coordinate NS_4_ coordination geometries are found in each of Zn(S_2_CNEt)_2_(pyOH) and Zn[S_2_CN(Me)CH_2_CH_2_OH]_2_(pyOH); pyOH is 3-hy­droxy­pyridine. In their respective crystals, hydrogen bonding leads to dimeric aggregates in the former (O—H⋯S) and supra­molecular chains in the latter (O—H⋯O, S).

## Chemical context   

The structures of binary zinc bis­(di­thio­carbamates) are always zero-dimensional (*i.e.* mol­ecular) (Heard, 2005 ▸) in contrast to their cadmium (Tan *et al.*, 2016*b* ▸) and mercury (Jotani *et al.*, 2016 ▸) analogues; di­thio­carbamate is ^−^S_2_CN*RR*’. The zinc structures can be mononuclear, distorted tetra­hedral as in Zn(S_2_CNCy_2_)_2_ (Cox & Tiekink, 2009 ▸) or, far more commonly, binuclear as in the archetypical compound [Zn(S_2_CNEt_2_)_2_]_2_, where heavily distorted five-coordinate geometries are found for zinc as two of the ligands are chelating and the others are μ_2_-tridentate (Bonamico *et al.*, 1965 ▸; Tiekink, 2000 ▸), with the adoption of one form over the other often being related to the steric bulk of the *R*/*R*′ groups (Tiekink, 2003 ▸). However, there is no clear-cut delineation between the adoption of one structural motif over the other depending on steric bulk. This is nicely illustrated in the structure of Zn[S_2_CN(*i*-Bu)_2_]_2_ which has equal numbers of both motifs (Ivanov *et al.*, 2005 ▸). A popular process by which structures of greater dimensionality might be formed is by the addition of neutral, potentially bridging ligands. However, in the case of zinc di­thio­carbamates, complexation with bidentate ligands usually results in the isolation of zero-dimensional, binuclear mol­ecules, *e.g*. {Zn[S_2_CN(Me)*i*-Pr)]_2_}_2_(Me_2_NCH_2_CH_2_NMe_2_) (Malik *et al.*, 1997 ▸); [Zn(S_2_CNMe_2_)_2_]_2_(4,4′-bipyrid­yl) (Zha *et al.*, 2010 ▸) and [Zn(S_2_CNEt_2_)_2_]_2_(Ph_2_PCH_2_CH_2_PPh_2_) (Zeng *et al.*, 1994 ▸). Even when excess base is included in the reaction, *e.g. trans*-1,2-bis­(4-pyrid­yl)ethyl­ene (bpe), only the zero-dimensional binuclear compound is isolated with non-coordinating bpe solvate, *i.e*. Zn(S_2_CNEt_2_)_2_]_2_(bpe)·bpe (Lai & Tiekink, 2003 ▸). That this reluctance to form coordination polymers is related directly to the nature of the di­thio­carbamate ligand is seen in the adoption of zigzag chains in analogous xanthate complexes, *e.g*. {[Zn(S_2_CO*R*)_2_]_2_(bpe)}_*n*_, for *R* = Et and *n*-Bu (Kang *et al.*, 2010 ▸). Steric effects come into play when *R* = Cy whereby a binuclear species is isolated, *i.e*. [Zn(S_2_COCy)_2_]_2_(bpe) (Kang *et al.*, 2010 ▸). This difference in chemistry arises to the significant (40%) contribution of the canonical structure ^(2-)^S_2_CN^(+)^ *RR*′, with two formally negatively charged sulfur atoms, which makes di­thio­carbamate a very effective chelating agent, thereby decreasing the Lewis acidity of the zinc atom.
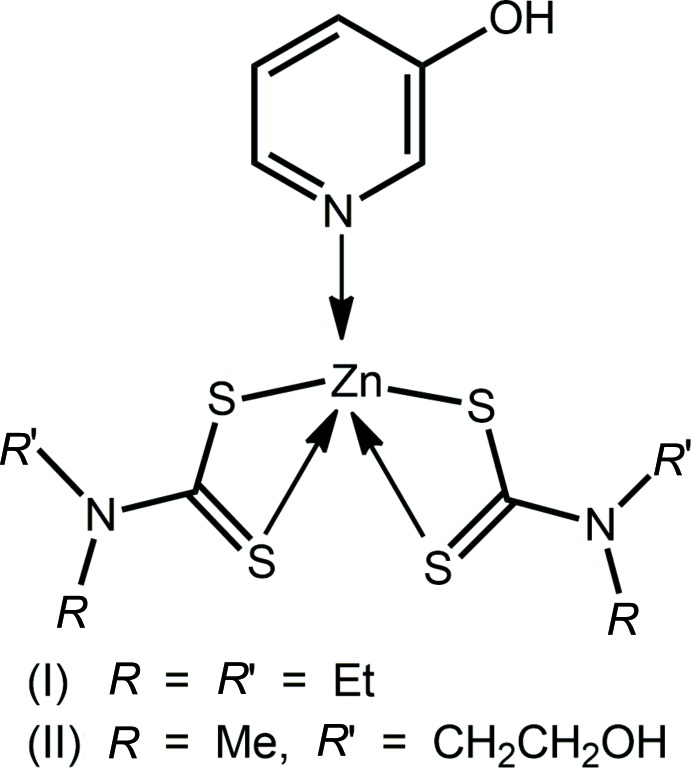


An approach to increase the supra­molecular aggregation in the crystal structures of zinc di­thio­carbamates has been to introduce hydrogen bonding functionality into the ligands, *i.e* using di­thio­carbamate anions of the type ^−^S_2_CN(*R*)CH_2_CH_2_OH. This influence is seen in the recent report of the structures of Zn[S_2_CN(*R*)CH_2_CH_2_OH]_2_(2,2′-bipyrid­yl) for *R* = *i*-Pr and CH_2_CH_2_OH (Safbri *et al.*, 2016 ▸). The common feature of these structures along with those of related species with no hydrogen bonding potential, *e.g*. Zn(S_2_CNMe_2_)_2_(2,2′-bipyrid­yl) (Manohar *et al.*, 1998 ▸), is the presence of a distorted octa­hedral N_2_S_4_ donor set about the zinc atom. The O—H⋯O hydrogen bonding in Zn[S_2_CN(*R*)CH_2_CH_2_OH]_2_(2,2′-bipyrid­yl), in the case when *R* = CH_2_CH_2_OH, isolated as a 1:1 hydrate, leads to supra­molecular ladders and these extend in two dimensions *via* water-O—H⋯S(di­thio­carbamate) hydrogen bonds. When *R* = *i*-Pr, layers are sustained by hy­droxy-O—H⋯S hydrogen bonds (Safbri *et al.*, 2016 ▸). As an extension of these studies, in the present report, Zn(S_2_CN*RR*′)_2_ has been complexed with 3-hy­droxy­pyridine (pyOH) to yield two 1:1 complexes. Quite different aggregation patterns are observed when *R* = *R*′ = Et (I), and *R* = *i*-Pr and *R*′ = CH_2_CH_2_OH (II). The crystal and mol­ecular structures of (I) and (II) are described herein along with an analysis of their Hirshfeld surfaces.

## Structural commentary   

**Figure 1:**
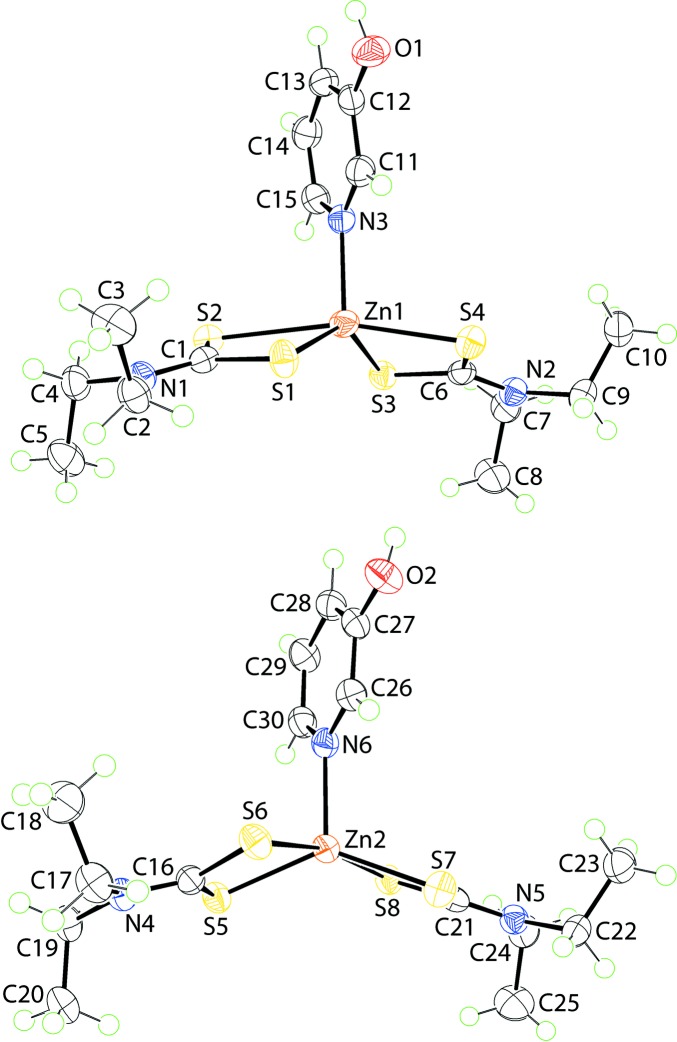
The mol­ecular structures of the two independent mol­ecules comprising the asymmetric unit in (I), showing the atom-labelling scheme and displacement ellipsoids at the 70% probability level.

Two independent mol­ecules of Zn(S_2_CNEt_2_)_2_(pyOH) comprise the asymmetric unit of (I), Fig. 1 ▸; pyOH is 3-hy­droxy­pyridine. For the Zn1-containing mol­ecule, Fig. 1 ▸ *a*, the Zn^II^ atom is chelated by two di­thio­carbamate ligands and one nitro­gen atom derived from the monodentate pyOH ligand. The S1-di­thio­carbamate ligand chelates the zinc atom forming quite different Zn—S bond lengths compared with the S3-di­thio­carbamate ligand. This is qu­anti­fied in the values of Δ(Zn—S), being the difference between the Zn—S_long_ and Zn—S_short_ bond lengths, Table 1 ▸, *i.e*. 0.43 and 0.15 Å, respectively. The Zn1—N3 bond length is significantly shorter than the Zn—S bonds. The NS_4_ coordination geometry is highly distorted as seen in the value of τ of 0.48 (Addison *et al.*, 1984 ▸). This value is almost exactly inter­mediate between the ideal square pyramidal geometry with τ = 0.0 and ideal trigonal pyramidal with τ = 1.0. The acute S—Zn—S chelate angles contribute to this distortion, Table 1 ▸. The widest angles in the coordination geometry are subtended by S_s_—Zn—S_s_ (s = short) and, especially, the S_l_—Zn—S_l_ (l = long) bond angles, Table 1 ▸. The coordination geometry for the Zn2 atom, Fig. 1 ▸ *b*, is quite similar to that just described for the Zn1 atom. The values of Δ(Zn—S) of 0.21 and 0.25 Å are inter­mediate to those for the Zn1-mol­ecule. Even so, the differences in the Zn—S bond lengths in both mol­ecules are not that great with this observation reflected in the closeness of the C—S bond lengths, Table 1 ▸. The value of τ for the Zn2-mol­ecule is 0.53, indicating an inclination towards trigonal bipyramidal *cf*. the Zn1-mol­ecule.

**Figure 2:**
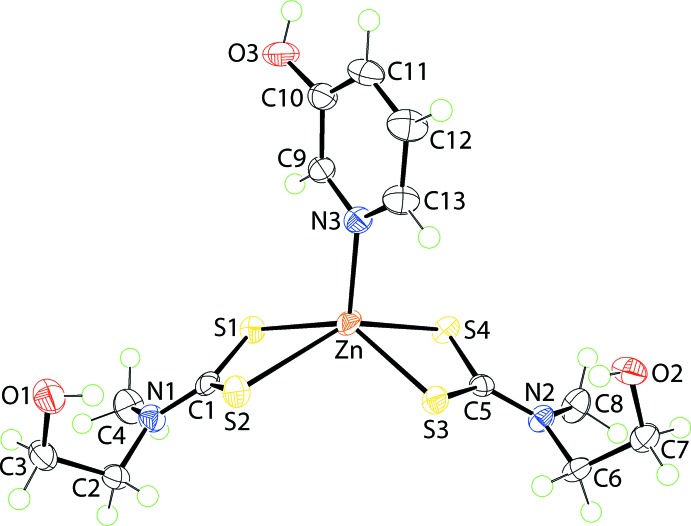
The mol­ecular structure of (II), showing the atom-labelling scheme and displacement ellipsoids at the 70% probability level.

The mol­ecular structure of (II), Zn[S_2_CN(Me)CH_2_CH_2_OH]_2_(pyOH), is shown in Fig. 2 ▸ and selected geometric parameters are included in Table 1 ▸. The coordination modes of the di­thio­carbamate ligands in (II) are close to those observed for the Zn1-mol­ecule in (I) with Δ(Zn—S) values of 0.42 and 0.19 Å. The difference between (I) and (II) is found in the coordination geometry which is close to square pyramidal in (II), as seen in the value of τ = 0.16. In this description, the S1–S4 atoms define the basal plane with the r.m.s. deviation being 0.0501 Å. The Zn atom lies 0.7514 (4) Å above the plane in the direction of the N3 atom. The dihedral angle between the chelate rings is 63.81 (15)°, an angle significantly greater than for the comparable angles in (I), Table 1 ▸.

**Figure 3:**
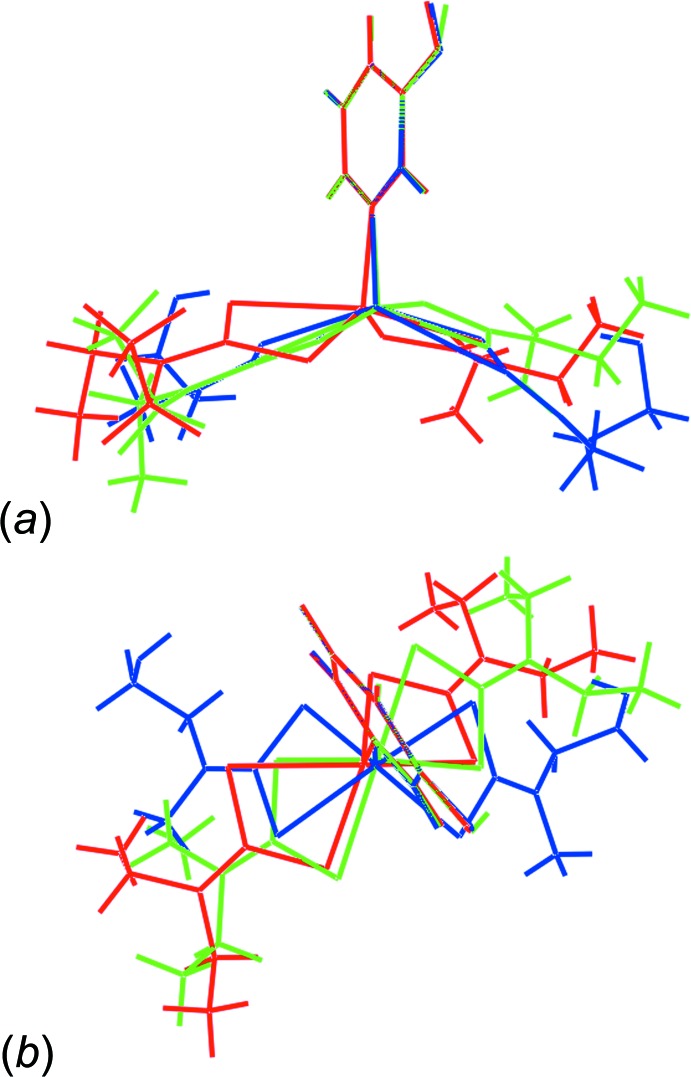
Overlay diagrams for the Zn1- and Zn2-mol­ecules in (I) and the mol­ecule in (II) shown as red, green and blue images, respectively: (*a*) approximately side-on to the pyOH ring and (*b*) along the N—Zn bond. The mol­ecules are overlapped so that the pyOH rings are coincident.

Overlay diagrams of the three mol­ecules in (I) and (II) are shown in Fig. 3 ▸. The mol­ecules have been overlapped so that the pyOH rings are coincident. The differences in the conformations of the mol­ecules comprising (I) are clearly seen, and especially between these and the conformation in (II). Such variability in structure reflects the flexibility in the binding modes of the di­thio­carbamate ligands leading to quite distinctive coordination geometries.

## Supra­molecular features   

**Figure 4:**
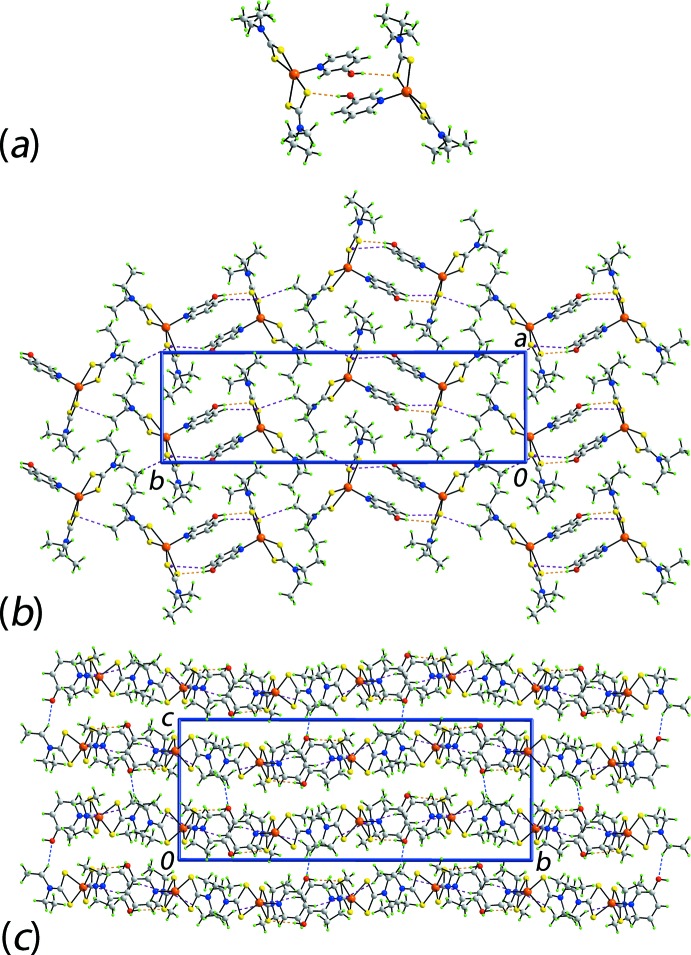
The mol­ecular packing in (I), showing (*a*) detail of the hy­droxy-O—H⋯S(di­thio­carbamate) hydrogen bonding, shown as orange dashed lines, leading to dimeric aggregates, (*b*) supra­molecular layer where the aggregates in (*a*) are linked by C—H⋯π(chelate) inter­actions, shown as purple dashed lines and (*c*) view of the unit-cell contents shown in projection down the *a* axis, with links between layers being of the type C—H⋯O, shown as blue dashed lines.

The key feature of the mol­ecular packing of (I) is the formation of hy­droxy-O—H⋯S(di­thio­carbamate) hydrogen bonds that sustain centrosymmetric, dimeric aggregates, *via* a 14-membered {⋯HOC_2_NZnS}_2_ synthon, Fig. 4 ▸ *a* and Table 2 ▸. Additional stabilization to the dimer is provided by an intra-dimer π–π inter­action between the pyOH rings. The inter-centroid distance is 3.5484 (18) Å and the angle of inclination is 3.91 (14)° for symmetry operation 1 − *x*, 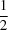 + *y*, 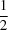 − *z*. The aggregates are further stabilized by pyOH-C—H⋯π inter­actions where the π-system is a chelate ring. Such C—H⋯π(chelate) inter­actions are increasingly being recognized as being important in the supra­molecular chemistry of metal 1,1-di­thiol­ates (Tiekink & Zukerman-Schpector, 2011 ▸; Tan *et al.*, 2016*a* ▸) and, it should be noted, routinely appear in the output from *PLATON* (Spek, 2009 ▸). Connections between aggregates leading to supra­molecular layers in the *ab* plane are also of the type C—H⋯π(chelate) but with methyl-H atoms as the donors, Fig. 4 ▸ *b*. The connections between layers along the *c* direction are of the type methyl­ene-C—H⋯O(hy­droxy), Fig. 4 ▸ *c*.

**Figure 5:**
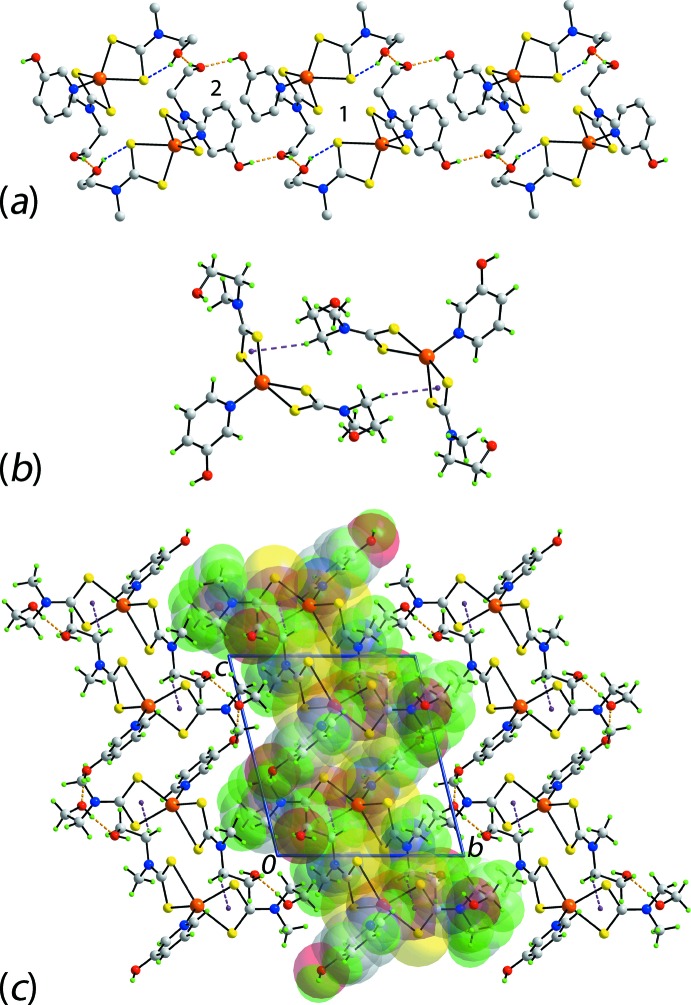
The mol­ecular packing in (II), (*a*) supra­molecular chain mediated by hy­droxy-O—H⋯O(hy­droxyl), S(dithiocarbamate) hydrogen bonding, shown as orange and blue dashed lines, respectively, and non-acidic H atoms omitted, (*b*) detail of methyl­ene-C—H⋯π(chelate) inter­actions shown as purple dashed lines and (*c*) view of the unit-cell contents shown in projection down the *a* axis, with one layer shown in space-filling mode.

The addition of greater hydrogen-bonding potential in (II) results in an infinite chain, Table 3 ▸. There is an hy­droxy-O—H⋯O(hy­droxy) hydrogen bond involving the O2 and O1 atoms as the donor and acceptor, respectively. The O1-hydroxy group forms a hydrogen bond with a di­thio­carbamate-S2 atom. As shown by the ‘1’ in Fig. 5 ▸ *a*, these hydrogen bonds lead to a centrosymmetric 22-membered {⋯SZnSCNC_2_OH⋯OH}_2_ synthon. On either side of these synthons, the pyOH hy­droxy group hydrogen bonds to the O2-hy­droxy atom and through symmetry, a centrosymmetric 24-membered {⋯OC_2_NCSZnNC_2_OH}_2_ synthon is formed, highlighted as ‘2’ in Fig. 5 ▸ *a*. Alternating synthons generate a supra­molecular chain aligned along the *c* axis. Methyl­ene-C—H⋯π(chelate) inter­actions link mol­ecules into dimeric units, Fig. 5 ▸ *b*. The combination of the aforementioned inter­actions lead to supra­molecular layers that stack along the *b* axis with no directional inter­actions between them, Fig. 5 ▸ *c*.

## Hirshfeld surface analysis   

**Figure 6:**
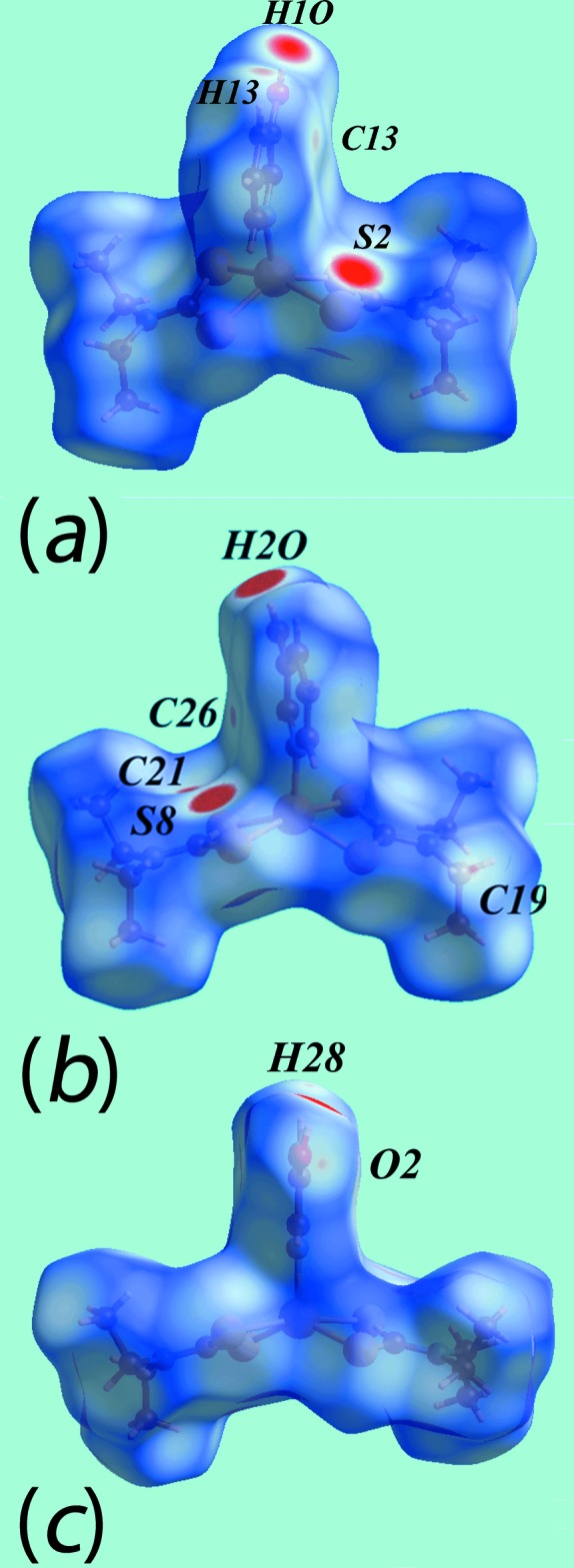
Views of the Hirshfeld surfaces for (I) mapped over *d*_norm_ for the (*a*) Zn1-mol­ecule and, (*b*) and (*c*) Zn2-mol­ecule.

**Figure 7:**
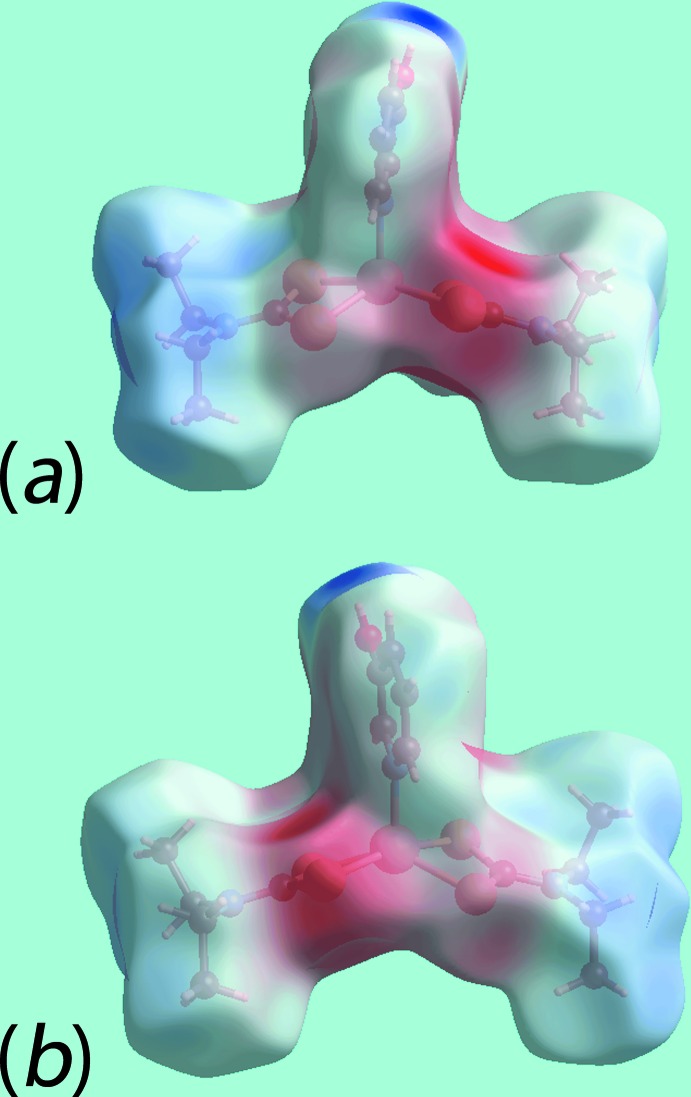
Views of the Hirshfeld surfaces mapped over electrostatic potential for (I): (*a*) Zn1-mol­ecule and (*b*) Zn2-mol­ecule.

**Figure 8:**
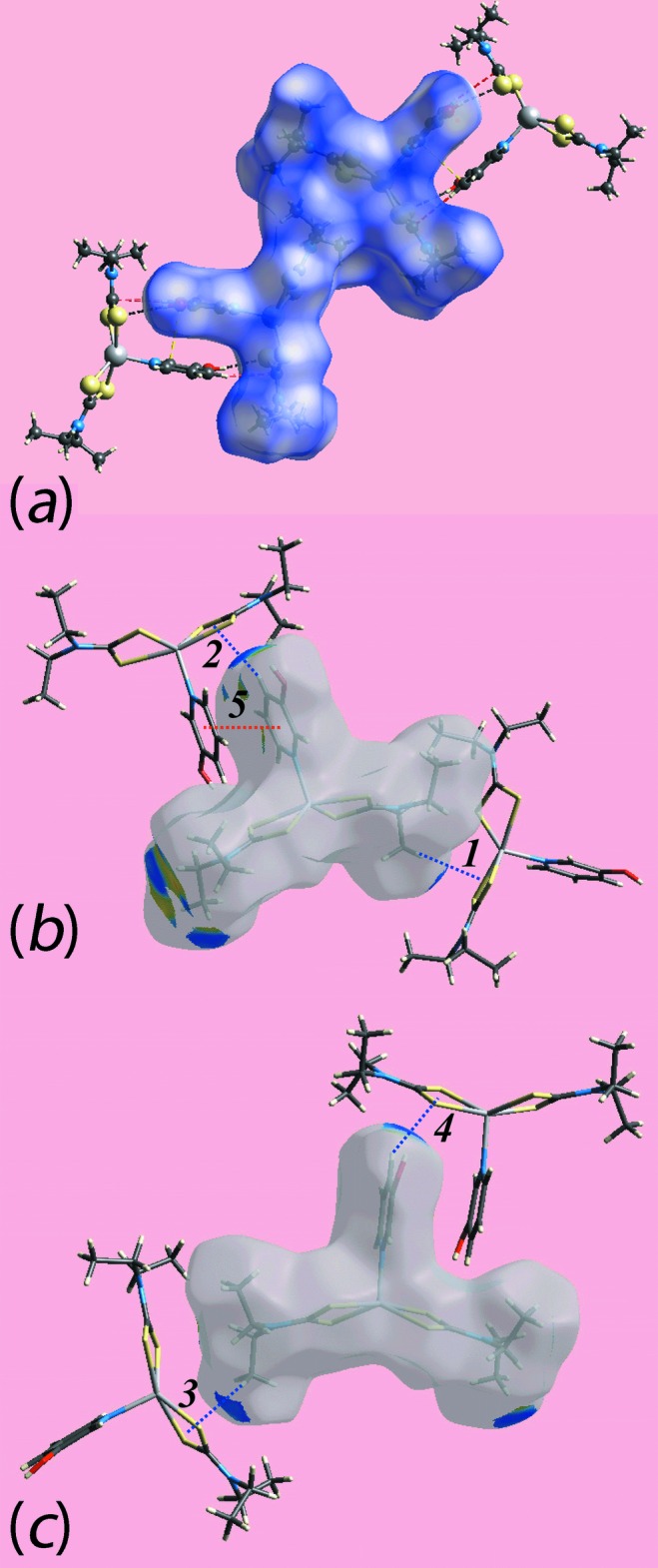
(*a*) View of the Hirshfeld surface mapped over *d*_norm_ for (I) showing O—H⋯S hydrogen bonds and short inter­atomic C⋯C and C⋯H/H⋯C contacts, indicated by black, white and red dashed lines, respectively, about the reference mol­ecule. (*b*) and (*c*) Views of Hirshfeld surface mapped with shape-index property about the Zn1 and Zn2-containing mol­ecules, respectively. The dotted blue lines labelled with 1-4 indicates C—H⋯π(chelate) inter­actions and the red dotted line shows the π–π stacking inter­action.

The Hirshfeld surface analysis for (I) and (II) was performed as described recently (Cardoso *et al.*, 2016 ▸). From the views of the Hirshfeld surface mapped over *d*_norm_ in the range −0.2 to + 1.3 au for the Zn1- and Zn2-containing mol­ecules of (I), Fig. 6 ▸, the presence of bright-red spots near the hy­droxy-H1*O* and -H2*O*, and di­thio­carbamate-S2 and S8 atoms represent the donors and acceptors of the O—H⋯S hydrogen bonds; these are viewed as blue and red regions on the Hirshfeld surfaces mapped over electrostatic potential (mapped over the range −0.07 to +0.10 au), Fig. 7 ▸, corresponding to positive and negative potentials, respectively. The faint-red spots appearing near the hy­droxy-O2 and methyl-C19 atoms in Fig. 6 ▸ *b* and 6*c* are due to comparatively weaker inter­molecular C—H⋯O inter­actions. The intra-dimer π–π stacking inter­action between the pyOH rings, Fig. 4 ▸ *a*, is evident through the appearance of faint-red spots near the arene-C13 and C26 atoms of the rings, Fig. 6 ▸ *a* and 6*b*, forming a close inter­atomic C⋯C contact, Table 4 ▸. The diminutive-red spots near the pyOH-H13 and -H28 and di­thio­carbamate-C21 atoms, Fig. 6 ▸ *a*–*c*, characterize the influence of the C—H⋯π(chelate) inter­actions; in Fig. 7 ▸, the light-blue and red regions represent the respective donors and acceptors for these inter­actions. The immediate environments around reference mol­ecules showing above inter­molecular inter­actions are illustrated in Fig. 8 ▸.

**Figure 9:**
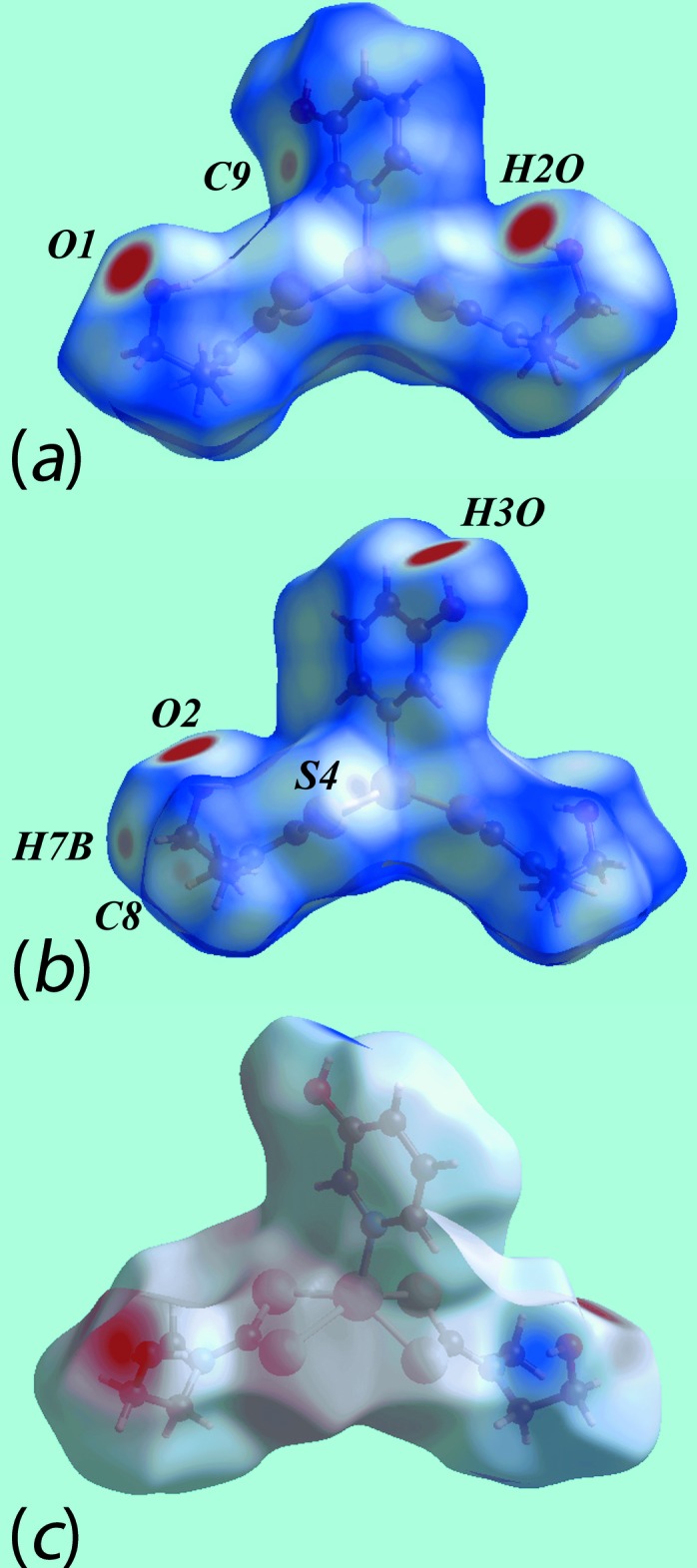
Views of the Hirshfeld surfaces for (II) mapped over (*a*) and (*b*) *d*_norm_ and (*c*) electrostatic potential.

**Figure 10:**
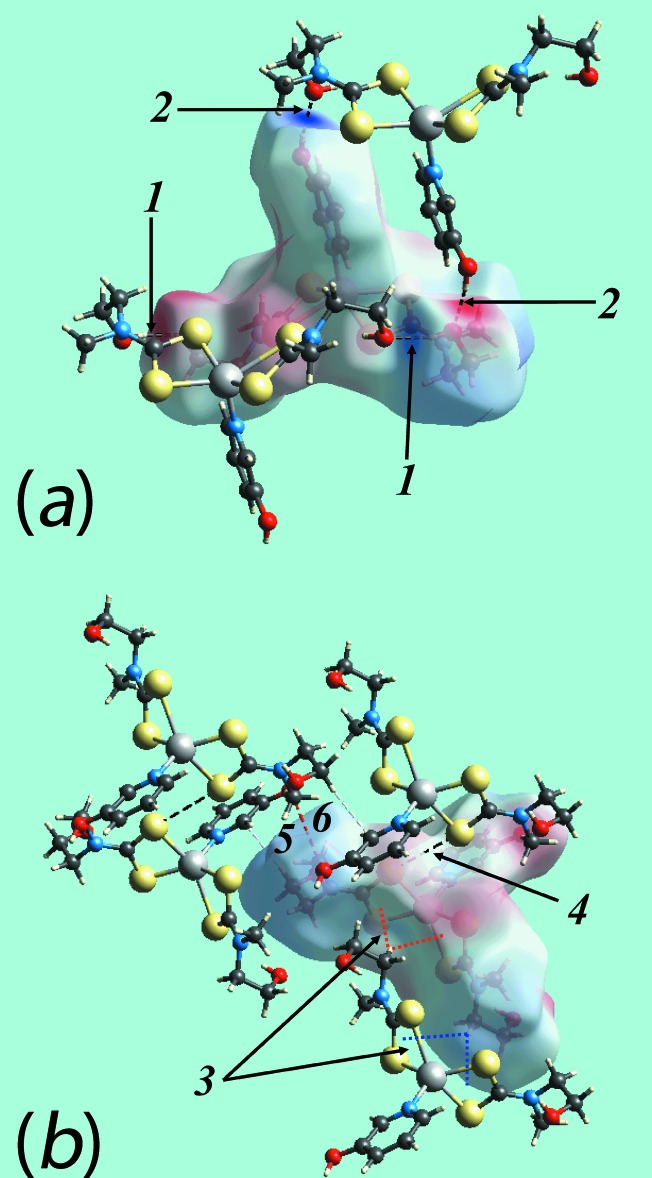
(*a*) and (*b*) Views of the Hirshfeld surface mapped over electrostatic potential for (II) showing O—H⋯S hydrogen bonds about the reference mol­ecule. The hydrogen bonds are indicated with black dashed lines and labelled as ‘1’ and ‘2’ in (*a*). In (*b*), the inter­molecular C—H⋯O (labelled with a ‘6’ and shown as red-dashed lines) and C—H⋯π/π⋯H—C (‘3’, red and blue) inter­actions, and short inter­atomic S⋯S (‘4’, black) and C⋯H (‘5’, white) contacts are indicated by arrows.

The presence of peripheral hy­droxy groups participating in the O—H⋯O hydrogen bonds in the structure of (II) result in the distinct bright-red spots near the respective donors and acceptor atoms on the Hirshfeld surface mapped over *d*_norm_, Fig. 9 ▸ *a* and 9*b*, and result in the blue and red regions corres­ponding to positive and negative potential on the Hirshfeld surface mapped over electrostatic potential (mapped over the range −0.12 to +0.18 au), Fig. 9 ▸ *c*. The faint-red spots near the S4, C8, C9 and H2*B* atoms in Fig. 9 ▸ *a* and 9*b* indicate their involvement in short inter­atomic S⋯S, C⋯C and C⋯H/H⋯C contacts, Table 4 ▸. Fig. 10 ▸ *a* illustrates the immediate environment about a reference mol­ecule within Hirshfeld surfaces mapped over electrostatic potential and highlights the O—H⋯O hydrogen bonds. The C—H⋯π(chelate) and its reciprocal contact, *i.e*. π—H⋯C, and short inter­atomic S⋯S, C⋯C and C⋯H/H⋯C contacts, with labels 3–6, are shown in Fig. 10 ▸ *b*.

**Figure 11:**
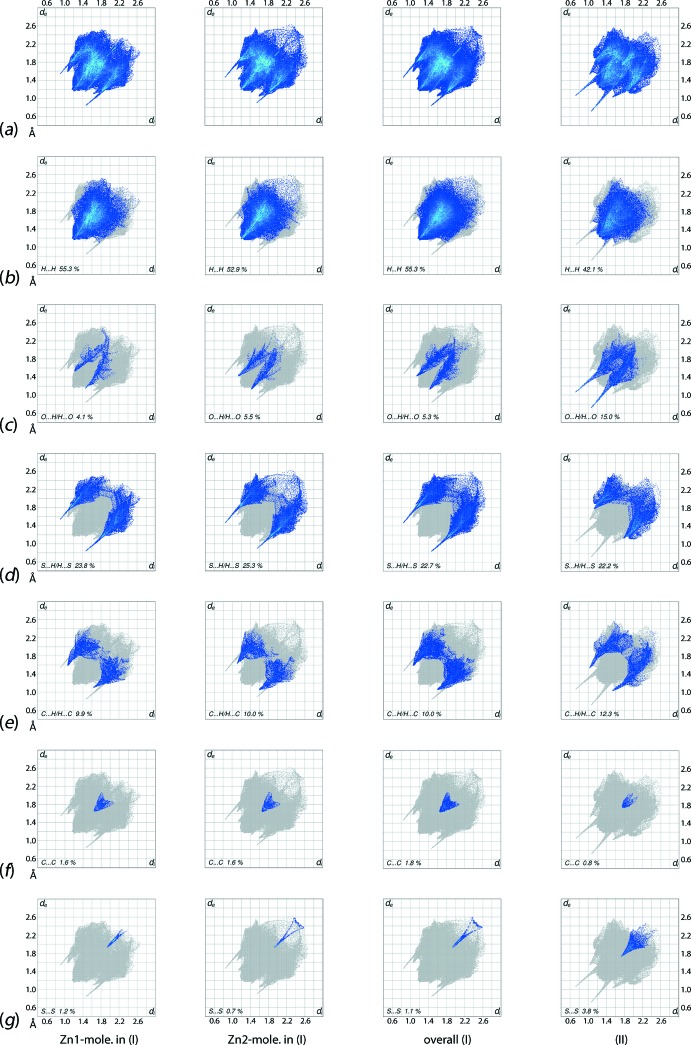
(*a*) The overall two-dimensional fingerprint plots for the Zn1-mol­ecule in (I), Zn2-mol­ecule in (I), (I) and (II), respectively, and those delineated into (*b*) H⋯H, (*c*) O⋯H/H⋯O, (*d*) S⋯H/H⋯S, (e) C⋯H/H⋯C, (f) C⋯C and (g) S⋯S contacts.

The overall two-dimensional fingerprint plot for individual Zn1- and Zn2-containing mol­ecules, overall (I) and (II) are illustrated in Fig. 11 ▸ *a*. The respective plots delineated into H⋯H, O⋯H/H⋯O, S⋯H/H⋯S, C⋯H/H⋯C, C⋯C and S⋯S contacts (McKinnon *et al.*, 2007 ▸) are shown in Fig. 11 ▸ *b*–*g*, respectively; the relative contributions from different contacts to the Hirshfeld surfaces of (I) and (II) are summarized in Table 5 ▸.

The fingerprint plots delineated into H⋯H contacts for (I), Fig. 11 ▸ *b*, show different distributions of points in the individ­ual plots for Zn1- and Zn2-mol­ecules. This, as well as their different percentage contributions to the Hirshfeld surface, Table 5 ▸, confirm their distinct chemical environments. The overall plot is the superimposition of these individual plots with a pair of small peaks, at (*d*_e_, *d*_i_) distances shorter than their van der Waals separations, corresponding to short inter­atomic H⋯H contacts, Table 4 ▸, between the hydrogen atoms of the Zn1-mol­ecule.

The fingerprint plots delineated into O⋯H/H⋯O contacts, Fig. 11 ▸ *c*, also exhibit slightly different profiles for the independent mol­ecules. The respective peaks at *d*_e_ + *d*_i_ ∼ 2.7 Å and ∼ 2.6 Å correspond to donors (upper region) and the acceptors (lower region) for the Zn1-mol­ecule, whereas these appear as a pair of peaks at the same *d*_e_ + *d*_i_ ∼ 2.6 Å distance for the Zn2-mol­ecule. This is likely due to the inter­acting oxygen and hydrogen atoms for the Zn1-mol­ecule being at their van der Waals separation in the donor region, *i.e*. at 2.72 Å, while in the acceptor region the peak corresponds to a short inter­atomic O⋯H contact, Table 4 ▸. In the plot for the Zn2-mol­ecule, this contact gives rise to the pair of peaks at *d*_e_ + *d*_i_ ∼ 2.6 Å.

The pair of spikes with their tips at different *d*_e_ + *d*_i_ distances in the fingerprint plots delineated into S⋯H/H⋯S contacts, Fig. 11 ▸ *d*, for the Zn1- and Zn2-mol­ecules result from different hy­droxy-O—H⋯S(di­thio­carbamate) hydrogen bonds. The tips at *d*_e_ + *d*_i_ ∼ 2.4 Å in the donor region of the plot for the Zn1-mol­ecule and in the acceptor region for the Zn2-mol­ecule are due to the formation of O—H⋯S hydrogen bonds between the hy­droxy-O1 and di­thio­carbamate-S8 atoms; the other hydrogen bond, involving the O2 and S2 atoms, gives rise to tips at *d*_e_ + *d*_i_ ∼ 2.3 Å in the respective donor and acceptor regions of the plots, Fig. 11 ▸ *d*. The plot for the overall structure results from the superimposition of individual plots and shows the symmetric distribution of points as a pair of long spikes having tips at *d*_e_ + *d*_i_ ∼ 2.3 Å. The short inter­atomic S⋯H/H⋯S contacts in the crystal of (I), Table 4 ▸, appear as a pair of aligned green points beginning at *d*_e_ + *d*_i_ ∼ 3.0 Å in the respective plots.

Almost the same percentage contribution from C⋯H/H⋯C contacts to the overall surface is made by the Zn1- and Zn2-mol­ecules, Table 5 ▸, and the respective fingerprint plots, Fig. 11 ▸ *e*, have the same shape with tips at *d*_e_ + *d*_i_ ∼ 2.7 Å which are due to the short inter­atomic C⋯H/H⋯C contacts, Table 4 ▸, involving the atoms forming the C—H⋯π(chelate) inter­actions; the points corresponding to the other short C⋯H/H⋯C contacts are within the plot. The C⋯C contacts assigned to intra-dimer π–π stacking inter­actions between pyOH-rings have a small, *i.e*. 1.8%, but recognizable contribution to the Hirshfeld surface and appear as an arrow-like distribution of points around *d*_e_ = *d*_i_ = 1.8 Å in Fig. 11 ▸ *f*. As indicated in Fig. 11 ▸ *g*, S⋯S contacts do not figure prominently in the mol­ecular packing of (I).

The corresponding two-dimensional fingerprint plots for (II) are also given in Fig. 11 ▸. In the fingerprint plots delineated into H⋯H contacts, Fig. 11 ▸ *b*, a pair of very thin spikes having their tips at *d*_e_ + *d*_i_ ∼ 2.3 Å indicate the presence of short inter­atomic H⋯H contacts between hy­droxy-H1*O* and -H2*O* atoms, Table 4 ▸. Also, the inter­molecular O—H⋯O hydrogen bond between the pyOH-O3 and hy­droxy-O2 atoms results in a short inter­atomic H⋯H contact between the H2*O* and H3*O* atoms, Table 4 ▸. The increase in the percentage contribution from O⋯H/H⋯O contacts to the Hirshfeld surface and the corresponding decrease in the contribution from H⋯H contacts in (II), *cf*. (I), Table 5 ▸, is due to the presence of dominating O—H⋯O hydrogen bonds in the crystal of (II) and is characterized as a pair of long spikes terminating at *d*_e_ + *d*_i_ ∼ 1.8 Å, Fig. 11 ▸ *c*. The tips corresponding to the O1⋯H6*A* contact, Table 4 ▸, are diminished within the long spikes corresponding to dominant O—H⋯O hydrogen bonds.

The S⋯H/H⋯S contacts with the nearly same contribution to the surface of (II) as for (I), *i.e*. 22.2 and 22.7%, respectively, reflect the O—H⋯S hydrogen bonds and additional S⋯H contacts resulting in tips at *d*_e_ + *d*_i_ ∼ 2.9 Å in Fig. 11 ▸ *d* and Table 4 ▸. The 12.3% contribution from C⋯H/H⋯C contacts to the surface with the tips at *d*_e_ + *d*_i_ ∼ 2.6 Å in the plot, Fig. 11 ▸ *e*, results from the C—H⋯π(chelate) and short inter­atomic C⋯H/H⋯C contacts, Table 4 ▸. The presence of C—H⋯π(chelate) inter­actions is also indicated by the short inter­atomic Zn⋯H/H⋯Zn contacts summarized in Table 4 ▸. The presence of short inter­atomic C⋯C contacts between symmetry-related methyl-C8 atoms is identified in the respective plot, Fig. 11 ▸ *f*, as the pair of tips at *d*_e_ + *d*_i_ ∼1.7 Å. Finally, a cone-shaped distribution of points with a 3.8% contribution to the surface from S⋯S contacts having a vertex at *d*_e_ = *d*_i_ ∼ 1.7 Å in the fingerprint plot, Fig. 11 ▸ *g*, results from short inter­atomic contacts between S4 atoms, Table 4 ▸; the absence of analogous contacts in (I) results in a very low percentage contribution to its surface (see above).

## Database survey   

As alluded to in the *Chemical context*, the presence of hydroxy­ethyl groups in zinc di­thio­carbamates leads to a higher degree of recognizable supra­molecular aggregation owing to hydrogen bonding, usually of the type hy­droxy-O—H⋯O(hy­droxy) but, sometimes also of the type hy­droxy-O—H⋯S(di­thio­carbamate) (Tan *et al.*, 2013 ▸; Jamaludin *et al.*, 2016 ▸). The following is a brief overview of some previous structures with ethyl­hydroxy­dithio­carbamate ligands highlighting the important role of hydrogen bonding in the supra­molecular aggregation. In the what might be termed the parent binary compound, *i.e*. {Zn[S_2_CN(CH_2_CH_2_OH)_2_]_2_}_2_, the usual dimeric motif is evident but these self-assemble *via* strong hydrogen bonding into three-dimensional architectures in both of the polymorphs characterized thus far, with the difference between the structures being the topology of supra­molecular layers, *i.e.* flattened (Manohar *et al.*, 1998 ▸) and undulating (Benson *et al.*, 2007 ▸). When one ethyl­hydroxy group is replaced by an ethyl group, as in {Zn[S_2_CN(Et)CH_2_CH_2_OH]_2_}_2_, the reduced hydrogen bonding leads to supra­molecular chains (Benson *et al.*, 2007 ▸). Bridging ligands lead to zero-dimensional aggregates, *e.g*. in {Zn[S_2_CN(Me)CH_2_CH_2_OH)_2_]_2_}_2_ *L*, where *L* is (3-pyrid­yl)CH_2_N(H)C(=O)C(=O)N(H)CH_2_(3-pyrid­yl). However, hydrogen bonding of the type hy­droxy-O—H⋯O(hy­droxy) links the mol­ecules into inter-woven double chains (Poplaukhin & Tiekink, 2008 ▸). The inter­esting structural chemistry is complimented by observations that some of these compounds exhibit exciting, cell-specific, anti-cancer potential (Tan *et al.*, 2015 ▸). The foregoing suggests this is a fertile area of research, well deserving of continuing attention.

## Synthesis and crystallization   

Synthesis of (I): In a 2:1:0.5 molar ratio, Zn(S_2_CNEt_2_)_2_, *N*,*N*′-bis­(pyridin-3-ylmeth­yl)ethane­dithiodi­amide (Zukerman-Schpector *et al.*, 2015 ▸) and 3-hy­droxy pyridine were dissolved in chloro­form. Solvent diffusion of hexane into this solution produced pink crystals. FT–IR (cm^−1^): ν(C=N) 1482 (*s*, *br*); ν(C—S) 987 (*s*). ^1^H NMR (*d*_6_-DMSO, 300 MHz): δ 9.91 (*s*, 1H, OH), 8.20–8.00 (*m*, 2H, aromatic-H), 7.30–7.10 (*m*, 2H, aromatic-H), 3.82 (8H, *q*, NCH_2_, *J* = 7.00 Hz); 1.22 (12H, *t*, CH_3_, *J* = 7.20 Hz).

Synthesis of (II): In a 1:1 molar ratio, Zn[S_2_N(Me)CH_2_CH_2_OH]_2_ and 3-hy­droxy pyridine were dissolved in a MeOH/EtOH (1:1 *v*/*v*) solution. Solvent diffusion of hexane into this solution led to the formation of colourless crystals. FT–IR (cm^−1^): ν(C=N) 1480 (*s*); ν(C—S) 1002 (*s*). ^1^H NMR (*d*_6_-DMSO, 300 MHz): δ 9.91 (*s*, 1H, aromatic-OH), 8.20–8.00 (*m*, 2H, aromatic-H), 7.30–7.10 (*m*, 2H, aromatic-H), 4.91 (2H, *t*, OH, *J* = 5.50 Hz); 3.90 (4H, *t*, NCH_2_, *J* = 6.25 Hz); 3.70 (4H, *dt*, CH_2_O, *J* = 5.50, 5.50 Hz); 3.41 (6H, *s*, CH_3_).

## Refinement details   

Crystal data, data collection and structure refinement details are summarized in Table 6 ▸. The carbon-bound H-atoms were placed in calculated positions (C—H = 0.95–0.99 Å) and were included in the refinement in the riding-model approximation, with *U*_iso_(H) set to 1.2–1.5*U*_eq_(C). The oxygen-bound H-atoms were located in difference Fourier maps but were refined with a distance restraint of O—H = 0.84±0.01 Å, and with *U*_iso_(H) set to 1.5*U*_eq_(O).

## Supplementary Material

Crystal structure: contains datablock(s) . DOI: 10.1107/S205698901601728X/hb7628sup1.cif

Structure factors: contains datablock(s) I. DOI: 10.1107/S205698901601728X/hb7628Isup2.hkl

Structure factors: contains datablock(s) II. DOI: 10.1107/S205698901601728X/hb7628IIsup3.hkl

CCDC references: 1511865, 1511864

Additional supporting information:  crystallographic information; 3D view; checkCIF report

## Figures and Tables

**Table 1:** Geometric data (Å, °) for (I) and (II)

Parameter	Zn1-mol­ecule in (I)	Zn2-mol­ecule in (I)	(II)
Zn—S1	2.3201 (8)	–	2.3319 (6)
Zn—S2	2.7461 (8)	–	2.7514 (8)
Zn—S3	2.3417 (8)	–	2.3437 (7)
Zn—S4	2.4932 (8)	–	2.5275 (6)
Zn—S5	–	2.3399 (8)	–
Zn—S6	–	2.5453 (8)	–
Zn—S7	–	2.3517 (8)	–
Zn—S8	–	2.6051 (8)	–
Zn—N3	2.069 (2)	–	2.0375 (16)
Zn—N6	–	2.070 (2)	–
C—S1, S2	1.736 (3), 1.721 (3)	–	1.733 (2), 1.7119 (19)
C—S3, S4	1.741 (3), 1.720 (3)	–	1.7364 (19), 1.7140 (19)
C—S5, S6	–	1.743 (3), 1.720 (3)	–
C—S7, S8	–	1.734 (3), 1.730 (3)	–
S1—Zn—S2	70.99 (3)	–	70.825 (18)
S3—Zn—S4	75.54 (3)	–	74.41 (2)
S1—Zn—S3	136.44 (3)	–	139.04 (2)
S2—Zn—S4	165.17 (2)	–	148.839 (18)
S5—Zn—S6	–	74.34 (3)	–
S7—Zn—S8	–	73.08 (3)	–
S5—Zn—S7	–	137.08 (3)	–
S6—Zn—S8	–	168.91 (2)	–
S1,S2,C1/S3,S4,*C*	19.30 (12)	–	63.81 (15)
S5,S6,C1/S7,S8,*C*	–	38.87 (22)	–

**Table 2:** Hydrogen-bond geometry (Å, °) for (I) *Cg*1 and *Cg*2 are the centroids of the (Zn1,S1,S2,C1) and (Zn2,S7,S8,C21) chelate rings, respectively.

*D*—H⋯*A*	*D*—H	H⋯*A*	*D*⋯*A*	*D*—H⋯*A*
O1—H1*O*⋯S8^i^	0.84 (2)	2.45 (1)	3.289 (2)	173 (4)
O2—H2*O*⋯S2^ii^	0.84 (2)	2.31 (1)	3.143 (2)	170 (4)
C8—H8*A*⋯*Cg*2	0.98	2.98	3.855 (3)	150
C13—H13⋯*Cg*2^i^	0.95	2.79	3.631 (3)	148
C20—H20*C*⋯*Cg*1^iii^	0.98	2.97	3.850 (3)	150
C28—H28⋯*Cg*1^ii^	0.95	2.96	3.738 (3)	140
C19—H19*A*⋯O2^iv^	0.99	2.56	3.321 (3)	134

Symmetry codes: (i) 
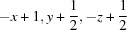
; (ii) 
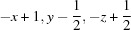
; (iii) 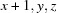; (iv) 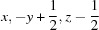.

**Table 3:** Hydrogen-bond geometry (Å, °) for (II) *Cg*1 is the centroid of the (Zn,S3,S4,C5) chelate ring.

*D*—H⋯*A*	*D*—H	H⋯*A*	*D*⋯*A*	*D*—H⋯*A*
O1—H1*O*⋯S2	0.84 (2)	2.61 (2)	3.371 (2)	152 (3)
O2—H2*O*⋯O1^i^	0.83 (3)	1.94 (3)	2.734 (2)	161 (3)
O3—H3*O*⋯O2^ii^	0.84 (3)	1.79 (2)	2.619 (2)	170 (3)
C2—H2*B*⋯*Cg*1^iii^	0.99	2.76	3.689 (2)	156

Symmetry codes: (i) 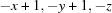; (ii) 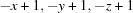; (iii) 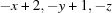.

**Table 4:** Summary of short inter­atomic contacts (Å) in (I) and (II)

Contact	Distance	Symmetry operation
(I)		
C13⋯C26	3.314 (4)	1 − *x*, 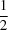 + *y*, 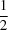 − *z*
H5⋯H7*B*	2.36	−*x*, 1 − *y*, −*z*
O1⋯H18*B*	2.61	2 − *x*, 1 − *y*, 1 − *z*
S2⋯H20*B*	2.96	1 − *x*, 1 − *y*, −*z*
S4⋯H11	2.98	1 − *x*, 1 − *y*, 1 − *z*
S5⋯H7*A*	2.97	*x*, *y*, *z*
S5⋯H14	2.94	1 − *x*, 1 − *y*, −*z*
C1⋯H28	2.75	1 − *x*, 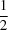 + *y*, 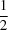 − *z*
C21⋯H13	2.65	1 − *x*, − 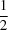 + *y*, 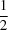 − *z*
C29⋯H24*A*	2.84	1 + *x*, *y*, *z*
(II)		
S4⋯S4	3.4765 (11)	2 − *x*, 1 − *y*, 1 − *z*
C8⋯C8	3.308 (3)	2 − *x*, −*y*, 1 − *z*
C1⋯H6*A*	2.87	*x*, 1 + *y*, *z*
C9⋯H7*B*	2.57	*x*, 1 + *y*, *z*
C10⋯H10*B*	2.88	*x*, 1 + *y*, *z*
H1*O*⋯H2*O*	2.37 (4)	1 − *x*, 1 − *y*, −*z*
H2*O*⋯H3*O*	2.18 (3)	1 − *x*, 1 − *y*, 1 − *z*
S3⋯H1*O*	2.91 (3)	1 − *x*, 1 − *y*, −*z*
S3⋯H7*A*	2.99	1 − *x*, 1 − *y*, −*z*
Zn⋯H2*B*	3.06	2 − *x*, 1 − *y*, −*z*
O1⋯H6*A*	2.68	*x*, 1 + *y*, *z*

**Table 5:** Percentage contribution to inter­atomic contacts from the Hirshfeld surface for (I) and (II)

Contact	Zn1-mol­ecule in (I)	Zn2-mol­ecule in (I)	(I)	(II)
H⋯H	55.3	52.9	55.3	42.1
O⋯H/H⋯O	4.1	5.5	5.3	15.0
S⋯H/H⋯S	23.8	25.3	22.7	22.2
C⋯H/H⋯C	9.9	10.0	10.0	12.3
N⋯H/H⋯N	2.6	2.5	2.7	2.9
S⋯S	1.2	0.7	1.1	3.8
C⋯C	1.6	1.6	1.8	0.8
Zn⋯H/H⋯Zn	0.8	0.8	0.4	0.7
C⋯O/O⋯C	0.4	0.4	0.4	0.0
C⋯N/N⋯C	0.2	0.2	0.3	0.1
S⋯O/O⋯S	0.1	0.1	0.0	0.0
S⋯C/C⋯S	0.0	0.0	0.0	0.1

**Table 6:** Experimental details

	(I)	(II)
Crystal data		
Chemical formula	[Zn(C_5_H_10_NS_2_)_2_(C_5_H_5_NO)]	[Zn(C_4_H_8_NOS_2_)_2_(C_5_H_5_NO)]
*M*_r_	456.99	460.94
Crystal system, space group	Monoclinic, *P*2_1_/*c*	Triclinic, *P* 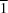
Temperature (K)	98	98
*a*, *b*, *c* (Å)	10.032 (2), 31.955 (7), 13.233 (3)	8.8645 (19), 9.956 (2), 11.473 (3)
α, β, γ (°)	90, 105.920 (2), 90	102.154 (4), 106.989 (4), 93.466 (3)
*V* (Å^3^)	4079.4 (15)	938.6 (4)
*Z*	8	2
Radiation type	Mo *K*α	Mo *K*α
μ (mm^−1^)	1.62	1.77
Crystal size (mm)	0.50 × 0.40 × 0.15	0.37 × 0.25 × 0.25

Data collection		
Diffractometer	Rigaku AFC12κ/SATURN724	Rigaku AFC12κ/SATURN724
Absorption correction	Multi-scan (*ABSCOR*; Higashi, 1995 ▸)	Multi-scan (*ABSCOR*; Higashi, 1995 ▸)
*T*_min_, *T*_max_	0.687, 1.000	0.860, 1.000
No. of measured, independent and observed [*I* > 2σ(*I*)] reflections	25139, 9202, 8401	6836, 4249, 4133
*R*_int_	0.037	0.026
(sin θ/λ)_max_ (Å^−1^)	0.650	0.650

Refinement		
*R*[*F* ^2^ > 2σ(*F* ^2^)], *wR*(*F* ^2^), *S*	0.041, 0.106, 1.06	0.032, 0.080, 1.06
No. of reflections	9202	4249
No. of parameters	447	228
No. of restraints	2	3
Δρ_max_, Δρ_min_ (e Å^−3^)	0.73, −0.45	0.43, −0.60

Computer programs: *CrystalClear* (Molecular Structure Corporation & Rigaku, 2005 ▸), *SHELXS97* (Sheldrick, 2008 ▸), *SHELXL2014* (Sheldrick, 2015 ▸), *ORTEP-3 for Windows* (Farrugia, 2012 ▸), *QMol* (Gans & Shalloway, 2001 ▸), *DIAMOND* (Brandenburg, 2006 ▸) and *publCIF* (Westrip, 2010 ▸).

## supplementary crystallographic information

### (I) Bis(N,N-diethyldithiocarbamato-κ2S,S')(3-hydroxypyridine-κN)zinc Crystal data

**Table:**

[Zn(C_5_H_10_NS_2_)_2_(C_5_H_5_NO)]	*F*(000) = 1904
*M**_r_* = 456.99	*D*_x_ = 1.488 Mg m^−^^3^
Monoclinic, *P*2_1_/*c*	Mo *K*α radiation, λ = 0.71073 Å
*a* = 10.032 (2) Å	Cell parameters from 16430 reflections
*b* = 31.955 (7) Å	θ = 2.5–40.7°
*c* = 13.233 (3) Å	µ = 1.62 mm^−^^1^
β = 105.920 (2)°	*T* = 98 K
*V* = 4079.4 (15) Å^3^	Slab, pink
*Z* = 8	0.50 × 0.40 × 0.15 mm

### (I) Bis(N,N-diethyldithiocarbamato-κ2S,S')(3-hydroxypyridine-κN)zinc Data collection

**Table:**

Rigaku AFC12κ/SATURN724 diffractometer	9202 independent reflections
Radiation source: fine-focus sealed tube	8401 reflections with *I* > 2σ(*I*)
Graphite monochromator	*R*_int_ = 0.037
ω scans	θ_max_ = 27.5°, θ_min_ = 2.5°
Absorption correction: multi-scan (ABSCOR; Higashi, 1995)	*h* = −10→13
*T*_min_ = 0.687, *T*_max_ = 1.000	*k* = −41→41
25139 measured reflections	*l* = −17→17

### (I) Bis(N,N-diethyldithiocarbamato-κ2S,S')(3-hydroxypyridine-κN)zinc Refinement

**Table:**

Refinement on *F*^2^	Primary atom site location: structure-invariant direct methods
Least-squares matrix: full	Secondary atom site location: difference Fourier map
*R*[*F*^2^ > 2σ(*F*^2^)] = 0.041	Hydrogen site location: mixed
*wR*(*F*^2^) = 0.106	*w* = 1/[σ^2^(*F*_o_^2^) + (0.0477*P*)^2^ + 4.2267*P*] where *P* = (*F*_o_^2^ + 2*F*_c_^2^)/3
*S* = 1.06	(Δ/σ)_max_ = 0.002
9202 reflections	Δρ_max_ = 0.73 e Å^−^^3^
447 parameters	Δρ_min_ = −0.45 e Å^−^^3^
2 restraints	

### (I) Bis(N,N-diethyldithiocarbamato-κ2S,S')(3-hydroxypyridine-κN)zinc Special details

**Table:**

Geometry. All esds (except the esd in the dihedral angle between two l.s. planes) are estimated using the full covariance matrix. The cell esds are taken into account individually in the estimation of esds in distances, angles and torsion angles; correlations between esds in cell parameters are only used when they are defined by crystal symmetry. An approximate (isotropic) treatment of cell esds is used for estimating esds involving l.s. planes.

### (I) Bis(N,N-diethyldithiocarbamato-κ2S,S')(3-hydroxypyridine-κN)zinc Fractional atomic coordinates and isotropic or equivalent isotropic displacement parameters (Å^2^)

**Table:**

	*x*	*y*	*z*	*U*_iso_*/*U*_eq_	
Zn1	0.22939 (3)	0.51145 (2)	0.27529 (3)	0.02233 (8)	
S1	0.06711 (7)	0.51485 (2)	0.37067 (5)	0.02470 (14)	
S2	−0.01500 (6)	0.54220 (2)	0.14827 (5)	0.02127 (13)	
S3	0.27656 (6)	0.46663 (2)	0.14937 (5)	0.02206 (13)	
S4	0.42106 (7)	0.46582 (2)	0.37705 (5)	0.02349 (14)	
O1	0.5087 (2)	0.64627 (6)	0.45759 (17)	0.0305 (4)	
H1O	0.524 (4)	0.6717 (4)	0.449 (3)	0.046*	
N1	−0.1829 (2)	0.54510 (7)	0.27436 (17)	0.0206 (4)	
N2	0.4941 (2)	0.41780 (7)	0.23507 (18)	0.0218 (4)	
N3	0.3333 (2)	0.56740 (7)	0.27551 (18)	0.0209 (4)	
C1	−0.0580 (3)	0.53538 (8)	0.2645 (2)	0.0194 (5)	
C2	−0.2190 (3)	0.54050 (9)	0.3748 (2)	0.0257 (5)	
H2A	−0.1631	0.5176	0.4162	0.031*	
H2B	−0.3180	0.5327	0.3602	0.031*	
C3	−0.1933 (3)	0.58063 (10)	0.4389 (2)	0.0339 (7)	
H3A	−0.0933	0.5856	0.4649	0.051*	
H3B	−0.2330	0.5780	0.4985	0.051*	
H3C	−0.2371	0.6041	0.3946	0.051*	
C4	−0.2961 (3)	0.55989 (9)	0.1855 (2)	0.0265 (5)	
H4A	−0.2567	0.5750	0.1350	0.032*	
H4B	−0.3553	0.5796	0.2114	0.032*	
C5	−0.3837 (3)	0.52333 (10)	0.1301 (2)	0.0334 (6)	
H5A	−0.3275	0.5054	0.0978	0.050*	
H5B	−0.4634	0.5340	0.0756	0.050*	
H5C	−0.4166	0.5071	0.1813	0.050*	
C6	0.4075 (3)	0.44668 (8)	0.2532 (2)	0.0203 (5)	
C7	0.4939 (3)	0.40561 (9)	0.1276 (2)	0.0268 (6)	
H7A	0.5897	0.3983	0.1272	0.032*	
H7B	0.4644	0.4299	0.0804	0.032*	
C8	0.3997 (3)	0.36900 (10)	0.0850 (3)	0.0353 (7)	
H8A	0.4218	0.3457	0.1350	0.053*	
H8B	0.4134	0.3602	0.0177	0.053*	
H8C	0.3030	0.3774	0.0748	0.053*	
C9	0.6003 (3)	0.39774 (8)	0.3208 (2)	0.0261 (5)	
H9A	0.6182	0.3691	0.2992	0.031*	
H9B	0.5651	0.3954	0.3836	0.031*	
C10	0.7353 (3)	0.42242 (10)	0.3491 (2)	0.0313 (6)	
H10A	0.7667	0.4266	0.2860	0.047*	
H10B	0.8060	0.4069	0.4015	0.047*	
H10C	0.7201	0.4497	0.3782	0.047*	
C11	0.3950 (3)	0.58787 (8)	0.3647 (2)	0.0236 (5)	
H11	0.4004	0.5749	0.4303	0.028*	
C12	0.4514 (3)	0.62776 (8)	0.3636 (2)	0.0236 (5)	
C13	0.4457 (3)	0.64633 (8)	0.2680 (2)	0.0242 (5)	
H13	0.4834	0.6734	0.2653	0.029*	
C14	0.3839 (3)	0.62468 (9)	0.1761 (2)	0.0266 (5)	
H14	0.3798	0.6366	0.1096	0.032*	
C15	0.3287 (3)	0.58556 (9)	0.1827 (2)	0.0233 (5)	
H15	0.2859	0.5709	0.1197	0.028*	
Zn2	0.68820 (3)	0.27217 (2)	0.19561 (3)	0.02217 (8)	
S5	0.75317 (6)	0.32449 (2)	0.09483 (5)	0.02196 (13)	
S6	0.89261 (7)	0.30961 (2)	0.31945 (5)	0.02480 (14)	
S7	0.52971 (7)	0.26483 (2)	0.29651 (5)	0.02464 (14)	
S8	0.45448 (6)	0.24765 (2)	0.06815 (5)	0.02173 (13)	
O2	0.9810 (2)	0.13960 (6)	0.36656 (16)	0.0293 (4)	
H2O	0.997 (4)	0.1141 (4)	0.358 (3)	0.044*	
N4	0.9764 (2)	0.36702 (7)	0.20279 (18)	0.0214 (4)	
N5	0.2806 (2)	0.23709 (7)	0.18746 (18)	0.0218 (4)	
N6	0.7917 (2)	0.21685 (7)	0.18654 (18)	0.0214 (4)	
C16	0.8854 (3)	0.33725 (8)	0.2066 (2)	0.0196 (5)	
C17	1.0857 (3)	0.37985 (9)	0.2973 (2)	0.0270 (6)	
H17A	1.0512	0.3764	0.3601	0.032*	
H17B	1.1075	0.4098	0.2914	0.032*	
C18	1.2165 (3)	0.35417 (10)	0.3110 (3)	0.0358 (7)	
H18A	1.1964	0.3247	0.3211	0.054*	
H18B	1.2880	0.3642	0.3725	0.054*	
H18C	1.2497	0.3570	0.2482	0.054*	
C19	0.9748 (3)	0.38995 (8)	0.1058 (2)	0.0246 (5)	
H19A	0.9319	0.3722	0.0442	0.030*	
H19B	1.0713	0.3959	0.1050	0.030*	
C20	0.8951 (3)	0.43075 (9)	0.0970 (2)	0.0284 (6)	
H20A	0.7968	0.4248	0.0881	0.043*	
H20B	0.9056	0.4466	0.0362	0.043*	
H20C	0.9315	0.4473	0.1610	0.043*	
C21	0.4063 (3)	0.24864 (8)	0.1840 (2)	0.0206 (5)	
C22	0.2413 (3)	0.23388 (9)	0.2868 (2)	0.0247 (5)	
H22A	0.1416	0.2405	0.2736	0.030*	
H22B	0.2948	0.2546	0.3377	0.030*	
C23	0.2691 (3)	0.19034 (10)	0.3332 (2)	0.0310 (6)	
H23A	0.2249	0.1696	0.2800	0.047*	
H23B	0.2311	0.1879	0.3937	0.047*	
H23C	0.3693	0.1853	0.3559	0.047*	
C24	0.1702 (3)	0.22577 (9)	0.0921 (2)	0.0262 (6)	
H24A	0.1135	0.2028	0.1086	0.031*	
H24B	0.2125	0.2158	0.0371	0.031*	
C25	0.0776 (3)	0.26349 (11)	0.0511 (3)	0.0371 (7)	
H25A	0.0353	0.2732	0.1053	0.056*	
H25B	0.0047	0.2554	−0.0118	0.056*	
H25C	0.1335	0.2860	0.0333	0.056*	
C26	0.8547 (3)	0.19591 (8)	0.2743 (2)	0.0225 (5)	
H26	0.8552	0.2078	0.3402	0.027*	
C27	0.9194 (3)	0.15755 (8)	0.2729 (2)	0.0223 (5)	
C28	0.9185 (3)	0.14053 (8)	0.1761 (2)	0.0249 (5)	
H28	0.9612	0.1143	0.1720	0.030*	
C29	0.8541 (3)	0.16261 (9)	0.0855 (2)	0.0261 (5)	
H29	0.8533	0.1517	0.0185	0.031*	
C30	0.7912 (3)	0.20054 (9)	0.0932 (2)	0.0242 (5)	
H30	0.7466	0.2154	0.0308	0.029*	

### (I) Bis(N,N-diethyldithiocarbamato-κ2S,S')(3-hydroxypyridine-κN)zinc Atomic displacement parameters (Å^2^)

**Table:**

	*U*^11^	*U*^22^	*U*^33^	*U*^12^	*U*^13^	*U*^23^
Zn1	0.02198 (15)	0.01845 (15)	0.02877 (17)	−0.00025 (11)	0.01071 (12)	−0.00238 (12)
S1	0.0212 (3)	0.0328 (3)	0.0206 (3)	0.0046 (2)	0.0065 (2)	0.0053 (3)
S2	0.0235 (3)	0.0222 (3)	0.0186 (3)	−0.0006 (2)	0.0066 (2)	−0.0003 (2)
S3	0.0209 (3)	0.0226 (3)	0.0217 (3)	0.0009 (2)	0.0042 (2)	−0.0024 (2)
S4	0.0270 (3)	0.0237 (3)	0.0202 (3)	0.0043 (2)	0.0073 (2)	−0.0009 (2)
O1	0.0391 (11)	0.0230 (10)	0.0248 (10)	−0.0026 (8)	0.0011 (9)	−0.0029 (8)
N1	0.0190 (10)	0.0225 (10)	0.0192 (11)	0.0006 (8)	0.0032 (8)	0.0007 (8)
N2	0.0232 (10)	0.0208 (10)	0.0222 (11)	0.0011 (8)	0.0074 (9)	−0.0022 (8)
N3	0.0192 (10)	0.0210 (10)	0.0222 (11)	0.0010 (8)	0.0052 (8)	0.0012 (8)
C1	0.0226 (12)	0.0163 (11)	0.0192 (12)	−0.0006 (9)	0.0054 (9)	−0.0002 (9)
C2	0.0212 (12)	0.0324 (14)	0.0251 (14)	0.0000 (10)	0.0090 (10)	0.0014 (11)
C3	0.0349 (15)	0.0411 (17)	0.0294 (15)	−0.0047 (13)	0.0148 (13)	−0.0096 (13)
C4	0.0208 (12)	0.0296 (14)	0.0270 (14)	0.0024 (10)	0.0031 (10)	0.0054 (11)
C5	0.0261 (13)	0.0419 (17)	0.0280 (15)	−0.0048 (12)	0.0002 (11)	0.0000 (13)
C6	0.0201 (11)	0.0168 (11)	0.0256 (13)	−0.0016 (9)	0.0088 (10)	0.0004 (10)
C7	0.0258 (13)	0.0278 (13)	0.0286 (14)	0.0034 (10)	0.0108 (11)	−0.0057 (11)
C8	0.0338 (15)	0.0347 (16)	0.0362 (17)	−0.0002 (12)	0.0075 (13)	−0.0166 (13)
C9	0.0273 (13)	0.0213 (12)	0.0293 (14)	0.0061 (10)	0.0069 (11)	0.0023 (11)
C10	0.0265 (13)	0.0316 (15)	0.0326 (16)	0.0047 (11)	0.0028 (12)	0.0020 (12)
C11	0.0240 (12)	0.0229 (12)	0.0226 (13)	0.0019 (10)	0.0040 (10)	0.0006 (10)
C12	0.0181 (11)	0.0239 (13)	0.0263 (14)	0.0028 (9)	0.0020 (10)	−0.0041 (10)
C13	0.0223 (12)	0.0202 (12)	0.0312 (15)	−0.0001 (9)	0.0090 (11)	0.0007 (10)
C14	0.0278 (13)	0.0280 (14)	0.0250 (14)	0.0036 (10)	0.0087 (11)	0.0039 (11)
C15	0.0218 (12)	0.0263 (13)	0.0211 (13)	0.0003 (10)	0.0045 (10)	−0.0001 (10)
Zn2	0.02096 (15)	0.01850 (15)	0.02802 (17)	−0.00040 (10)	0.00836 (12)	0.00219 (11)
S5	0.0212 (3)	0.0211 (3)	0.0224 (3)	−0.0027 (2)	0.0039 (2)	0.0019 (2)
S6	0.0299 (3)	0.0234 (3)	0.0208 (3)	−0.0021 (2)	0.0064 (3)	0.0019 (2)
S7	0.0225 (3)	0.0297 (3)	0.0209 (3)	−0.0035 (2)	0.0046 (2)	−0.0031 (3)
S8	0.0221 (3)	0.0229 (3)	0.0200 (3)	−0.0008 (2)	0.0055 (2)	0.0026 (2)
O2	0.0397 (11)	0.0231 (10)	0.0245 (10)	0.0017 (8)	0.0078 (9)	0.0027 (8)
N4	0.0217 (10)	0.0207 (10)	0.0210 (11)	−0.0033 (8)	0.0043 (8)	−0.0025 (8)
N5	0.0224 (10)	0.0221 (10)	0.0209 (11)	0.0000 (8)	0.0059 (9)	0.0010 (9)
N6	0.0186 (10)	0.0204 (10)	0.0247 (11)	−0.0032 (8)	0.0053 (8)	0.0005 (9)
C16	0.0222 (11)	0.0186 (11)	0.0195 (12)	0.0002 (9)	0.0083 (9)	−0.0007 (9)
C17	0.0295 (13)	0.0257 (13)	0.0222 (13)	−0.0072 (10)	0.0010 (11)	−0.0053 (10)
C18	0.0297 (14)	0.0371 (16)	0.0357 (17)	−0.0021 (12)	0.0008 (13)	−0.0001 (13)
C19	0.0286 (13)	0.0251 (13)	0.0223 (13)	−0.0056 (10)	0.0104 (11)	0.0005 (10)
C20	0.0348 (14)	0.0229 (13)	0.0280 (15)	−0.0049 (11)	0.0094 (12)	0.0033 (11)
C21	0.0228 (12)	0.0173 (11)	0.0203 (12)	0.0024 (9)	0.0038 (10)	0.0031 (9)
C22	0.0224 (12)	0.0294 (13)	0.0241 (14)	0.0006 (10)	0.0097 (10)	0.0002 (11)
C23	0.0300 (14)	0.0353 (15)	0.0291 (15)	0.0021 (12)	0.0102 (12)	0.0061 (12)
C24	0.0191 (12)	0.0310 (14)	0.0252 (14)	−0.0042 (10)	0.0006 (10)	−0.0010 (11)
C25	0.0282 (14)	0.0408 (17)	0.0348 (17)	0.0039 (12)	−0.0037 (13)	0.0059 (14)
C26	0.0235 (12)	0.0221 (12)	0.0214 (13)	−0.0022 (9)	0.0052 (10)	−0.0016 (10)
C27	0.0241 (12)	0.0186 (12)	0.0242 (13)	−0.0030 (9)	0.0068 (10)	0.0014 (10)
C28	0.0284 (13)	0.0197 (12)	0.0288 (14)	0.0000 (10)	0.0113 (11)	−0.0013 (10)
C29	0.0335 (14)	0.0259 (13)	0.0200 (13)	−0.0018 (11)	0.0091 (11)	−0.0006 (10)
C30	0.0244 (12)	0.0252 (13)	0.0227 (13)	−0.0014 (10)	0.0063 (10)	0.0038 (10)

### (I) Bis(N,N-diethyldithiocarbamato-κ2S,S')(3-hydroxypyridine-κN)zinc Geometric parameters (Å, º)

**Table:**

Zn1—N3	2.069 (2)	Zn2—N6	2.070 (2)
Zn1—S1	2.3201 (8)	Zn2—S5	2.3399 (8)
Zn1—S3	2.3417 (8)	Zn2—S7	2.3517 (8)
Zn1—S4	2.4932 (8)	Zn2—S6	2.5453 (8)
Zn1—S2	2.7461 (8)	Zn2—S8	2.6051 (8)
S1—C1	1.736 (3)	S5—C16	1.743 (3)
S2—C1	1.721 (3)	S6—C16	1.720 (3)
S3—C6	1.741 (3)	S7—C21	1.734 (3)
S4—C6	1.720 (3)	S8—C21	1.730 (3)
O1—C12	1.355 (3)	O2—C27	1.352 (3)
O1—H1O	0.842 (10)	O2—H2O	0.844 (10)
N1—C1	1.332 (3)	N4—C16	1.328 (3)
N1—C4	1.470 (3)	N4—C19	1.474 (3)
N1—C2	1.477 (3)	N4—C17	1.478 (3)
N2—C6	1.333 (3)	N5—C21	1.326 (3)
N2—C9	1.473 (3)	N5—C22	1.476 (3)
N2—C7	1.474 (3)	N5—C24	1.478 (3)
N3—C11	1.343 (3)	N6—C26	1.339 (3)
N3—C15	1.347 (3)	N6—C30	1.340 (4)
C2—C3	1.520 (4)	C17—C18	1.515 (4)
C2—H2A	0.9900	C17—H17A	0.9900
C2—H2B	0.9900	C17—H17B	0.9900
C3—H3A	0.9800	C18—H18A	0.9800
C3—H3B	0.9800	C18—H18B	0.9800
C3—H3C	0.9800	C18—H18C	0.9800
C4—C5	1.524 (4)	C19—C20	1.517 (4)
C4—H4A	0.9900	C19—H19A	0.9900
C4—H4B	0.9900	C19—H19B	0.9900
C5—H5A	0.9800	C20—H20A	0.9800
C5—H5B	0.9800	C20—H20B	0.9800
C5—H5C	0.9800	C20—H20C	0.9800
C7—C8	1.512 (4)	C22—C23	1.515 (4)
C7—H7A	0.9900	C22—H22A	0.9900
C7—H7B	0.9900	C22—H22B	0.9900
C8—H8A	0.9800	C23—H23A	0.9800
C8—H8B	0.9800	C23—H23B	0.9800
C8—H8C	0.9800	C23—H23C	0.9800
C9—C10	1.523 (4)	C24—C25	1.528 (4)
C9—H9A	0.9900	C24—H24A	0.9900
C9—H9B	0.9900	C24—H24B	0.9900
C10—H10A	0.9800	C25—H25A	0.9800
C10—H10B	0.9800	C25—H25B	0.9800
C10—H10C	0.9800	C25—H25C	0.9800
C11—C12	1.397 (4)	C26—C27	1.390 (4)
C11—H11	0.9500	C26—H26	0.9500
C12—C13	1.384 (4)	C27—C28	1.389 (4)
C13—C14	1.388 (4)	C28—C29	1.389 (4)
C13—H13	0.9500	C28—H28	0.9500
C14—C15	1.380 (4)	C29—C30	1.383 (4)
C14—H14	0.9500	C29—H29	0.9500
C15—H15	0.9500	C30—H30	0.9500
			
N3—Zn1—S1	112.77 (6)	N6—Zn2—S5	110.78 (6)
N3—Zn1—S3	109.24 (6)	N6—Zn2—S7	112.06 (6)
S1—Zn1—S3	136.44 (3)	S5—Zn2—S7	137.08 (3)
N3—Zn1—S4	101.02 (6)	N6—Zn2—S6	96.34 (6)
S1—Zn1—S4	106.61 (3)	S5—Zn2—S6	74.34 (3)
S3—Zn1—S4	75.54 (3)	S7—Zn2—S6	103.42 (3)
N3—Zn1—S2	93.23 (6)	N6—Zn2—S8	94.71 (6)
S1—Zn1—S2	70.99 (3)	S5—Zn2—S8	100.83 (3)
S3—Zn1—S2	95.97 (3)	S7—Zn2—S8	73.08 (3)
S4—Zn1—S2	165.17 (2)	S6—Zn2—S8	168.91 (2)
C1—S1—Zn1	92.11 (9)	C16—S5—Zn2	87.12 (9)
C1—S2—Zn1	78.96 (9)	C16—S6—Zn2	81.24 (9)
C6—S3—Zn1	85.32 (9)	C21—S7—Zn2	88.69 (9)
C6—S4—Zn1	81.11 (9)	C21—S8—Zn2	80.88 (9)
C12—O1—H1O	110 (3)	C27—O2—H2O	110 (3)
C1—N1—C4	122.5 (2)	C16—N4—C19	123.1 (2)
C1—N1—C2	122.3 (2)	C16—N4—C17	121.7 (2)
C4—N1—C2	115.2 (2)	C19—N4—C17	115.3 (2)
C6—N2—C9	122.1 (2)	C21—N5—C22	122.6 (2)
C6—N2—C7	121.9 (2)	C21—N5—C24	122.4 (2)
C9—N2—C7	116.0 (2)	C22—N5—C24	115.0 (2)
C11—N3—C15	118.9 (2)	C26—N6—C30	119.1 (2)
C11—N3—Zn1	122.19 (18)	C26—N6—Zn2	120.10 (18)
C15—N3—Zn1	118.67 (18)	C30—N6—Zn2	120.70 (18)
N1—C1—S2	122.1 (2)	N4—C16—S6	122.3 (2)
N1—C1—S1	119.98 (19)	N4—C16—S5	120.4 (2)
S2—C1—S1	117.91 (14)	S6—C16—S5	117.23 (14)
N1—C2—C3	111.8 (2)	N4—C17—C18	111.6 (2)
N1—C2—H2A	109.2	N4—C17—H17A	109.3
C3—C2—H2A	109.2	C18—C17—H17A	109.3
N1—C2—H2B	109.2	N4—C17—H17B	109.3
C3—C2—H2B	109.2	C18—C17—H17B	109.3
H2A—C2—H2B	107.9	H17A—C17—H17B	108.0
C2—C3—H3A	109.5	C17—C18—H18A	109.5
C2—C3—H3B	109.5	C17—C18—H18B	109.5
H3A—C3—H3B	109.5	H18A—C18—H18B	109.5
C2—C3—H3C	109.5	C17—C18—H18C	109.5
H3A—C3—H3C	109.5	H18A—C18—H18C	109.5
H3B—C3—H3C	109.5	H18B—C18—H18C	109.5
N1—C4—C5	110.8 (2)	N4—C19—C20	111.9 (2)
N1—C4—H4A	109.5	N4—C19—H19A	109.2
C5—C4—H4A	109.5	C20—C19—H19A	109.2
N1—C4—H4B	109.5	N4—C19—H19B	109.2
C5—C4—H4B	109.5	C20—C19—H19B	109.2
H4A—C4—H4B	108.1	H19A—C19—H19B	107.9
C4—C5—H5A	109.5	C19—C20—H20A	109.5
C4—C5—H5B	109.5	C19—C20—H20B	109.5
H5A—C5—H5B	109.5	H20A—C20—H20B	109.5
C4—C5—H5C	109.5	C19—C20—H20C	109.5
H5A—C5—H5C	109.5	H20A—C20—H20C	109.5
H5B—C5—H5C	109.5	H20B—C20—H20C	109.5
N2—C6—S4	122.2 (2)	N5—C21—S8	121.7 (2)
N2—C6—S3	120.0 (2)	N5—C21—S7	121.0 (2)
S4—C6—S3	117.86 (14)	S8—C21—S7	117.32 (15)
N2—C7—C8	113.5 (2)	N5—C22—C23	111.1 (2)
N2—C7—H7A	108.9	N5—C22—H22A	109.4
C8—C7—H7A	108.9	C23—C22—H22A	109.4
N2—C7—H7B	108.9	N5—C22—H22B	109.4
C8—C7—H7B	108.9	C23—C22—H22B	109.4
H7A—C7—H7B	107.7	H22A—C22—H22B	108.0
C7—C8—H8A	109.5	C22—C23—H23A	109.5
C7—C8—H8B	109.5	C22—C23—H23B	109.5
H8A—C8—H8B	109.5	H23A—C23—H23B	109.5
C7—C8—H8C	109.5	C22—C23—H23C	109.5
H8A—C8—H8C	109.5	H23A—C23—H23C	109.5
H8B—C8—H8C	109.5	H23B—C23—H23C	109.5
N2—C9—C10	111.8 (2)	N5—C24—C25	110.5 (2)
N2—C9—H9A	109.3	N5—C24—H24A	109.5
C10—C9—H9A	109.3	C25—C24—H24A	109.5
N2—C9—H9B	109.3	N5—C24—H24B	109.5
C10—C9—H9B	109.3	C25—C24—H24B	109.5
H9A—C9—H9B	107.9	H24A—C24—H24B	108.1
C9—C10—H10A	109.5	C24—C25—H25A	109.5
C9—C10—H10B	109.5	C24—C25—H25B	109.5
H10A—C10—H10B	109.5	H25A—C25—H25B	109.5
C9—C10—H10C	109.5	C24—C25—H25C	109.5
H10A—C10—H10C	109.5	H25A—C25—H25C	109.5
H10B—C10—H10C	109.5	H25B—C25—H25C	109.5
N3—C11—C12	121.7 (3)	N6—C26—C27	122.7 (3)
N3—C11—H11	119.2	N6—C26—H26	118.7
C12—C11—H11	119.2	C27—C26—H26	118.7
O1—C12—C13	123.6 (3)	O2—C27—C28	124.4 (2)
O1—C12—C11	117.4 (3)	O2—C27—C26	117.3 (2)
C13—C12—C11	119.1 (3)	C28—C27—C26	118.3 (3)
C12—C13—C14	118.9 (3)	C27—C28—C29	118.7 (3)
C12—C13—H13	120.5	C27—C28—H28	120.6
C14—C13—H13	120.5	C29—C28—H28	120.6
C15—C14—C13	119.1 (3)	C30—C29—C28	119.7 (3)
C15—C14—H14	120.5	C30—C29—H29	120.2
C13—C14—H14	120.5	C28—C29—H29	120.2
N3—C15—C14	122.3 (3)	N6—C30—C29	121.5 (3)
N3—C15—H15	118.8	N6—C30—H30	119.3
C14—C15—H15	118.8	C29—C30—H30	119.3
			
C4—N1—C1—S2	3.9 (3)	C19—N4—C16—S6	177.68 (19)
C2—N1—C1—S2	−178.13 (19)	C17—N4—C16—S6	−3.3 (3)
C4—N1—C1—S1	−175.75 (19)	C19—N4—C16—S5	−1.8 (3)
C2—N1—C1—S1	2.2 (3)	C17—N4—C16—S5	177.20 (19)
Zn1—S2—C1—N1	179.1 (2)	Zn2—S6—C16—N4	−177.2 (2)
Zn1—S2—C1—S1	−1.28 (12)	Zn2—S6—C16—S5	2.33 (12)
Zn1—S1—C1—N1	−178.9 (2)	Zn2—S5—C16—N4	177.0 (2)
Zn1—S1—C1—S2	1.49 (14)	Zn2—S5—C16—S6	−2.51 (13)
C1—N1—C2—C3	92.4 (3)	C16—N4—C17—C18	89.7 (3)
C4—N1—C2—C3	−89.5 (3)	C19—N4—C17—C18	−91.2 (3)
C1—N1—C4—C5	91.2 (3)	C16—N4—C19—C20	94.5 (3)
C2—N1—C4—C5	−86.9 (3)	C17—N4—C19—C20	−84.6 (3)
C9—N2—C6—S4	5.1 (3)	C22—N5—C21—S8	174.11 (19)
C7—N2—C6—S4	−171.71 (19)	C24—N5—C21—S8	−4.8 (3)
C9—N2—C6—S3	−175.58 (19)	C22—N5—C21—S7	−5.2 (3)
C7—N2—C6—S3	7.6 (3)	C24—N5—C21—S7	175.93 (19)
Zn1—S4—C6—N2	175.6 (2)	Zn2—S8—C21—N5	−177.8 (2)
Zn1—S4—C6—S3	−3.74 (12)	Zn2—S8—C21—S7	1.50 (12)
Zn1—S3—C6—N2	−175.4 (2)	Zn2—S7—C21—N5	177.7 (2)
Zn1—S3—C6—S4	3.94 (13)	Zn2—S7—C21—S8	−1.64 (14)
C6—N2—C7—C8	−91.5 (3)	C21—N5—C22—C23	−90.3 (3)
C9—N2—C7—C8	91.5 (3)	C24—N5—C22—C23	88.7 (3)
C6—N2—C9—C10	−88.5 (3)	C21—N5—C24—C25	−95.2 (3)
C7—N2—C9—C10	88.5 (3)	C22—N5—C24—C25	85.8 (3)
C15—N3—C11—C12	1.5 (4)	C30—N6—C26—C27	−0.4 (4)
Zn1—N3—C11—C12	−172.77 (19)	Zn2—N6—C26—C27	176.51 (19)
N3—C11—C12—O1	178.6 (2)	N6—C26—C27—O2	179.3 (2)
N3—C11—C12—C13	−1.2 (4)	N6—C26—C27—C28	0.2 (4)
O1—C12—C13—C14	−179.7 (2)	O2—C27—C28—C29	−178.6 (2)
C11—C12—C13—C14	0.0 (4)	C26—C27—C28—C29	0.4 (4)
C12—C13—C14—C15	0.8 (4)	C27—C28—C29—C30	−0.8 (4)
C11—N3—C15—C14	−0.7 (4)	C26—N6—C30—C29	0.1 (4)
Zn1—N3—C15—C14	173.8 (2)	Zn2—N6—C30—C29	−176.88 (19)
C13—C14—C15—N3	−0.4 (4)	C28—C29—C30—N6	0.6 (4)

### (I) Bis(N,N-diethyldithiocarbamato-κ2S,S')(3-hydroxypyridine-κN)zinc Hydrogen-bond geometry (Å, º)

Cg1 and Cg2 are the centroids of the (Zn1,S1,S2,C1) and (Zn2,S7,S8,C21) chelate rings, respectively.

**Table:**

*D*—H···*A*	*D*—H	H···*A*	*D*···*A*	*D*—H···*A*
O1—H1*O*···S8^i^	0.84 (2)	2.45 (1)	3.289 (2)	173 (4)
O2—H2*O*···S2^ii^	0.84 (2)	2.31 (1)	3.143 (2)	170 (4)
C8—H8*A*···*Cg*2	0.98	2.98	3.855 (3)	150
C13—H13···*Cg*2^i^	0.95	2.79	3.631 (3)	148
C20—H20*C*···*Cg*1^iii^	0.98	2.97	3.850 (3)	150
C28—H28···*Cg*1^ii^	0.95	2.96	3.738 (3)	140
C19—H19*A*···O2^iv^	0.99	2.56	3.321 (3)	134

Symmetry codes: (i) −*x*+1, *y*+1/2, −*z*+1/2; (ii) −*x*+1, *y*−1/2, −*z*+1/2; (iii) *x*+1, *y*, *z*; (iv) *x*, −*y*+1/2, *z*−1/2.

### (II) Bis[N-(2-hydroxyethyl)-N-methyldithiocarbamato-κ2S,S'](3-hydroxypyridine-κN)zinc Crystal data

**Table:**

[Zn(C_4_H_8_NOS_2_)_2_(C_5_H_5_NO)]	*Z* = 2
*M**_r_* = 460.94	*F*(000) = 476
Triclinic, *P*1	*D*_x_ = 1.631 Mg m^−^^3^
*a* = 8.8645 (19) Å	Mo *K*α radiation, λ = 0.71073 Å
*b* = 9.956 (2) Å	Cell parameters from 4145 reflections
*c* = 11.473 (3) Å	θ = 2.5–40.6°
α = 102.154 (4)°	µ = 1.77 mm^−^^1^
β = 106.989 (4)°	*T* = 98 K
γ = 93.466 (3)°	Slab, colourless
*V* = 938.6 (4) Å^3^	0.37 × 0.25 × 0.25 mm

### (II) Bis[N-(2-hydroxyethyl)-N-methyldithiocarbamato-κ2S,S'](3-hydroxypyridine-κN)zinc Data collection

**Table:**

Rigaku AFC12κ/SATURN724 diffractometer	4249 independent reflections
Radiation source: fine-focus sealed tube	4133 reflections with *I* > 2σ(*I*)
Graphite monochromator	*R*_int_ = 0.026
ω scans	θ_max_ = 27.5°, θ_min_ = 2.4°
Absorption correction: multi-scan (ABSCOR; Higashi, 1995)	*h* = −11→11
*T*_min_ = 0.860, *T*_max_ = 1.000	*k* = −12→12
6836 measured reflections	*l* = −14→14

### (II) Bis[N-(2-hydroxyethyl)-N-methyldithiocarbamato-κ2S,S'](3-hydroxypyridine-κN)zinc Refinement

**Table:**

Refinement on *F*^2^	Primary atom site location: structure-invariant direct methods
Least-squares matrix: full	Secondary atom site location: difference Fourier map
*R*[*F*^2^ > 2σ(*F*^2^)] = 0.032	Hydrogen site location: mixed
*wR*(*F*^2^) = 0.080	*w* = 1/[σ^2^(*F*_o_^2^) + (0.037*P*)^2^ + 0.6872*P*] where *P* = (*F*_o_^2^ + 2*F*_c_^2^)/3
*S* = 1.06	(Δ/σ)_max_ = 0.001
4249 reflections	Δρ_max_ = 0.43 e Å^−^^3^
228 parameters	Δρ_min_ = −0.60 e Å^−^^3^
3 restraints	

### (II) Bis[N-(2-hydroxyethyl)-N-methyldithiocarbamato-κ2S,S'](3-hydroxypyridine-κN)zinc Special details

**Table:**

Geometry. All esds (except the esd in the dihedral angle between two l.s. planes) are estimated using the full covariance matrix. The cell esds are taken into account individually in the estimation of esds in distances, angles and torsion angles; correlations between esds in cell parameters are only used when they are defined by crystal symmetry. An approximate (isotropic) treatment of cell esds is used for estimating esds involving l.s. planes.

### (II) Bis[N-(2-hydroxyethyl)-N-methyldithiocarbamato-κ2S,S'](3-hydroxypyridine-κN)zinc Fractional atomic coordinates and isotropic or equivalent isotropic displacement parameters (Å^2^)

**Table:**

	*x*	*y*	*z*	*U*_iso_*/*U*_eq_	
Zn	0.76357 (3)	0.49975 (2)	0.25271 (2)	0.01863 (8)	
S1	0.97385 (5)	0.65936 (5)	0.26357 (4)	0.01674 (11)	
S2	0.69910 (6)	0.58080 (5)	0.03011 (5)	0.01835 (11)	
S3	0.64889 (6)	0.27205 (5)	0.14638 (4)	0.01739 (11)	
S4	0.90377 (6)	0.34805 (5)	0.38990 (4)	0.01782 (11)	
O1	0.74043 (18)	0.85049 (17)	−0.09808 (15)	0.0260 (3)	
H1O	0.695 (3)	0.783 (2)	−0.083 (3)	0.039*	
O2	0.45844 (17)	0.01078 (16)	0.24736 (13)	0.0218 (3)	
H2O	0.413 (3)	0.051 (3)	0.193 (2)	0.033*	
O3	0.67339 (19)	0.91603 (16)	0.57620 (15)	0.0285 (3)	
H3O	0.624 (3)	0.945 (3)	0.627 (2)	0.043*	
N1	0.98304 (19)	0.70602 (17)	0.04700 (15)	0.0176 (3)	
N2	0.78664 (19)	0.08741 (16)	0.26777 (15)	0.0161 (3)	
N3	0.62518 (19)	0.59446 (17)	0.35049 (15)	0.0169 (3)	
C1	0.8924 (2)	0.65371 (18)	0.10539 (18)	0.0149 (3)	
C2	0.9350 (2)	0.6846 (2)	−0.09003 (18)	0.0191 (4)	
H2A	0.8502	0.6046	−0.1285	0.023*	
H2B	1.0271	0.6612	−0.1190	0.023*	
C3	0.8749 (2)	0.8097 (2)	−0.1355 (2)	0.0221 (4)	
H3A	0.9619	0.8882	−0.1017	0.027*	
H3B	0.8450	0.7881	−0.2282	0.027*	
C4	1.1446 (2)	0.7770 (2)	0.1154 (2)	0.0252 (4)	
H4A	1.1454	0.8372	0.1951	0.038*	
H4B	1.1789	0.8330	0.0647	0.038*	
H4C	1.2175	0.7081	0.1321	0.038*	
C5	0.7800 (2)	0.22057 (19)	0.26907 (17)	0.0143 (3)	
C6	0.6826 (2)	−0.02451 (19)	0.16625 (18)	0.0181 (4)	
H6A	0.7468	−0.0971	0.1421	0.022*	
H6B	0.6353	0.0126	0.0920	0.022*	
C7	0.5502 (2)	−0.0887 (2)	0.20439 (18)	0.0193 (4)	
H7A	0.4796	−0.1595	0.1316	0.023*	
H7B	0.5972	−0.1359	0.2718	0.023*	
C8	0.8976 (2)	0.0451 (2)	0.3718 (2)	0.0232 (4)	
H8A	1.0069	0.0797	0.3796	0.035*	
H8B	0.8852	−0.0562	0.3554	0.035*	
H8C	0.8751	0.0837	0.4499	0.035*	
C9	0.6861 (2)	0.7161 (2)	0.43240 (18)	0.0175 (4)	
H9	0.7921	0.7530	0.4436	0.021*	
C10	0.6007 (2)	0.7911 (2)	0.50204 (18)	0.0194 (4)	
C11	0.4471 (2)	0.7342 (2)	0.48828 (19)	0.0226 (4)	
H11	0.3855	0.7818	0.5347	0.027*	
C12	0.3863 (2)	0.6063 (2)	0.4053 (2)	0.0249 (4)	
H12	0.2826	0.5646	0.3953	0.030*	
C13	0.4768 (2)	0.5400 (2)	0.33723 (19)	0.0221 (4)	
H13	0.4331	0.4534	0.2794	0.027*	

### (II) Bis[N-(2-hydroxyethyl)-N-methyldithiocarbamato-κ2S,S'](3-hydroxypyridine-κN)zinc Atomic displacement parameters (Å^2^)

**Table:**

	*U*^11^	*U*^22^	*U*^33^	*U*^12^	*U*^13^	*U*^23^
Zn	0.02010 (13)	0.01232 (12)	0.02810 (14)	0.00449 (9)	0.01447 (10)	0.00421 (9)
S1	0.0155 (2)	0.0171 (2)	0.0178 (2)	0.00248 (17)	0.00462 (17)	0.00523 (17)
S2	0.0154 (2)	0.0194 (2)	0.0201 (2)	0.00046 (17)	0.00437 (18)	0.00651 (18)
S3	0.0217 (2)	0.0157 (2)	0.0149 (2)	0.00533 (17)	0.00362 (18)	0.00587 (17)
S4	0.0180 (2)	0.0143 (2)	0.0184 (2)	0.00276 (16)	0.00247 (18)	0.00227 (17)
O1	0.0257 (8)	0.0311 (8)	0.0317 (8)	0.0137 (6)	0.0166 (7)	0.0160 (7)
O2	0.0232 (7)	0.0268 (8)	0.0184 (7)	0.0090 (6)	0.0094 (6)	0.0064 (6)
O3	0.0326 (8)	0.0227 (8)	0.0321 (8)	0.0011 (6)	0.0208 (7)	−0.0037 (6)
N1	0.0157 (7)	0.0191 (8)	0.0198 (8)	0.0025 (6)	0.0077 (6)	0.0058 (6)
N2	0.0165 (7)	0.0140 (7)	0.0165 (7)	0.0040 (6)	0.0024 (6)	0.0043 (6)
N3	0.0157 (7)	0.0179 (8)	0.0192 (7)	0.0071 (6)	0.0063 (6)	0.0062 (6)
C1	0.0160 (8)	0.0107 (8)	0.0193 (8)	0.0053 (6)	0.0071 (7)	0.0037 (7)
C2	0.0236 (10)	0.0199 (9)	0.0181 (9)	0.0071 (7)	0.0114 (8)	0.0058 (7)
C3	0.0250 (10)	0.0261 (10)	0.0232 (9)	0.0091 (8)	0.0140 (8)	0.0118 (8)
C4	0.0167 (9)	0.0301 (11)	0.0295 (11)	−0.0018 (8)	0.0078 (8)	0.0093 (9)
C5	0.0151 (8)	0.0149 (8)	0.0154 (8)	0.0046 (6)	0.0076 (7)	0.0038 (7)
C6	0.0217 (9)	0.0125 (8)	0.0177 (8)	0.0037 (7)	0.0053 (7)	−0.0004 (7)
C7	0.0215 (9)	0.0157 (9)	0.0195 (9)	0.0036 (7)	0.0047 (7)	0.0038 (7)
C8	0.0228 (10)	0.0181 (9)	0.0257 (10)	0.0065 (8)	−0.0005 (8)	0.0091 (8)
C9	0.0176 (9)	0.0180 (9)	0.0195 (9)	0.0059 (7)	0.0077 (7)	0.0066 (7)
C10	0.0221 (9)	0.0212 (10)	0.0173 (9)	0.0065 (8)	0.0080 (8)	0.0060 (7)
C11	0.0204 (9)	0.0311 (11)	0.0196 (9)	0.0094 (8)	0.0101 (8)	0.0058 (8)
C12	0.0139 (9)	0.0333 (12)	0.0256 (10)	0.0040 (8)	0.0061 (8)	0.0026 (9)
C13	0.0177 (9)	0.0264 (10)	0.0201 (9)	0.0037 (8)	0.0053 (8)	0.0016 (8)

### (II) Bis[N-(2-hydroxyethyl)-N-methyldithiocarbamato-κ2S,S'](3-hydroxypyridine-κN)zinc Geometric parameters (Å, º)

**Table:**

Zn—N3	2.0375 (16)	C2—H2A	0.9900
Zn—S1	2.3319 (6)	C2—H2B	0.9900
Zn—S3	2.3437 (7)	C3—H3A	0.9900
Zn—S4	2.5275 (6)	C3—H3B	0.9900
Zn—S2	2.7514 (8)	C4—H4A	0.9800
S1—C1	1.733 (2)	C4—H4B	0.9800
S2—C1	1.7119 (19)	C4—H4C	0.9800
S3—C5	1.7364 (19)	C6—C7	1.518 (3)
S4—C5	1.7140 (19)	C6—H6A	0.9900
O1—C3	1.433 (2)	C6—H6B	0.9900
O1—H1O	0.833 (10)	C7—H7A	0.9900
O2—C7	1.418 (2)	C7—H7B	0.9900
O2—H2O	0.833 (10)	C8—H8A	0.9800
O3—C10	1.350 (2)	C8—H8B	0.9800
O3—H3O	0.834 (10)	C8—H8C	0.9800
N1—C1	1.333 (2)	C9—C10	1.393 (3)
N1—C4	1.468 (2)	C9—H9	0.9500
N1—C2	1.468 (2)	C10—C11	1.394 (3)
N2—C5	1.328 (2)	C11—C12	1.387 (3)
N2—C8	1.464 (2)	C11—H11	0.9500
N2—C6	1.466 (2)	C12—C13	1.379 (3)
N3—C9	1.337 (3)	C12—H12	0.9500
N3—C13	1.345 (3)	C13—H13	0.9500
C2—C3	1.516 (3)		
			
N3—Zn—S1	109.72 (5)	N1—C4—H4B	109.5
N3—Zn—S3	110.80 (5)	H4A—C4—H4B	109.5
S1—Zn—S3	139.04 (2)	N1—C4—H4C	109.5
N3—Zn—S4	103.07 (5)	H4A—C4—H4C	109.5
S1—Zn—S4	102.00 (2)	H4B—C4—H4C	109.5
S3—Zn—S4	74.41 (2)	N2—C5—S4	121.34 (14)
N3—Zn—S2	107.89 (5)	N2—C5—S3	121.19 (14)
S1—Zn—S2	70.825 (18)	S4—C5—S3	117.46 (11)
S3—Zn—S2	91.20 (2)	N2—C6—C7	112.02 (16)
S4—Zn—S2	148.839 (18)	N2—C6—H6A	109.2
C1—S1—Zn	90.54 (6)	C7—C6—H6A	109.2
C1—S2—Zn	77.85 (7)	N2—C6—H6B	109.2
C5—S3—Zn	86.67 (6)	C7—C6—H6B	109.2
C5—S4—Zn	81.43 (7)	H6A—C6—H6B	107.9
C3—O1—H1O	109 (2)	O2—C7—C6	112.50 (16)
C7—O2—H2O	113 (2)	O2—C7—H7A	109.1
C10—O3—H3O	110 (2)	C6—C7—H7A	109.1
C1—N1—C4	121.53 (17)	O2—C7—H7B	109.1
C1—N1—C2	122.52 (16)	C6—C7—H7B	109.1
C4—N1—C2	115.68 (16)	H7A—C7—H7B	107.8
C5—N2—C8	120.74 (16)	N2—C8—H8A	109.5
C5—N2—C6	122.91 (16)	N2—C8—H8B	109.5
C8—N2—C6	116.33 (15)	H8A—C8—H8B	109.5
C9—N3—C13	118.73 (17)	N2—C8—H8C	109.5
C9—N3—Zn	118.04 (13)	H8A—C8—H8C	109.5
C13—N3—Zn	123.22 (14)	H8B—C8—H8C	109.5
N1—C1—S2	122.54 (15)	N3—C9—C10	122.79 (18)
N1—C1—S1	118.66 (14)	N3—C9—H9	118.6
S2—C1—S1	118.79 (11)	C10—C9—H9	118.6
N1—C2—C3	113.44 (16)	O3—C10—C9	116.64 (18)
N1—C2—H2A	108.9	O3—C10—C11	125.07 (18)
C3—C2—H2A	108.9	C9—C10—C11	118.27 (19)
N1—C2—H2B	108.9	C12—C11—C10	118.52 (19)
C3—C2—H2B	108.9	C12—C11—H11	120.7
H2A—C2—H2B	107.7	C10—C11—H11	120.7
O1—C3—C2	112.64 (16)	C13—C12—C11	119.77 (19)
O1—C3—H3A	109.1	C13—C12—H12	120.1
C2—C3—H3A	109.1	C11—C12—H12	120.1
O1—C3—H3B	109.1	N3—C13—C12	121.87 (19)
C2—C3—H3B	109.1	N3—C13—H13	119.1
H3A—C3—H3B	107.8	C12—C13—H13	119.1
N1—C4—H4A	109.5		
			
C4—N1—C1—S2	175.51 (15)	Zn—S4—C5—S3	−1.66 (9)
C2—N1—C1—S2	−10.8 (2)	Zn—S3—C5—N2	−176.68 (15)
C4—N1—C1—S1	−4.7 (2)	Zn—S3—C5—S4	1.77 (10)
C2—N1—C1—S1	169.02 (14)	C5—N2—C6—C7	−103.9 (2)
Zn—S2—C1—N1	167.26 (16)	C8—N2—C6—C7	74.4 (2)
Zn—S2—C1—S1	−12.55 (9)	N2—C6—C7—O2	55.4 (2)
Zn—S1—C1—N1	−165.30 (14)	C13—N3—C9—C10	−2.0 (3)
Zn—S1—C1—S2	14.52 (10)	Zn—N3—C9—C10	178.55 (14)
C1—N1—C2—C3	102.8 (2)	N3—C9—C10—O3	−176.43 (17)
C4—N1—C2—C3	−83.2 (2)	N3—C9—C10—C11	2.1 (3)
N1—C2—C3—O1	−58.9 (2)	O3—C10—C11—C12	177.9 (2)
C8—N2—C5—S4	1.6 (3)	C9—C10—C11—C12	−0.5 (3)
C6—N2—C5—S4	179.87 (14)	C10—C11—C12—C13	−1.1 (3)
C8—N2—C5—S3	−179.97 (15)	C9—N3—C13—C12	0.2 (3)
C6—N2—C5—S3	−1.7 (3)	Zn—N3—C13—C12	179.67 (16)
Zn—S4—C5—N2	176.79 (16)	C11—C12—C13—N3	1.3 (3)

### (II) Bis[N-(2-hydroxyethyl)-N-methyldithiocarbamato-κ2S,S'](3-hydroxypyridine-κN)zinc Hydrogen-bond geometry (Å, º)

Cg1 is the centroid of the (Zn,S3,S4,C5) chelate ring.

**Table:**

*D*—H···*A*	*D*—H	H···*A*	*D*···*A*	*D*—H···*A*
O1—H1*O*···S2	0.84 (2)	2.61 (2)	3.371 (2)	152 (3)
O2—H2*O*···O1^i^	0.83 (3)	1.94 (3)	2.734 (2)	161 (3)
O3—H3*O*···O2^ii^	0.84 (3)	1.79 (2)	2.619 (2)	170 (3)
C2—H2*B*···*Cg*1^iii^	0.99	2.76	3.689 (2)	156

Symmetry codes: (i) −*x*+1, −*y*+1, −*z*; (ii) −*x*+1, −*y*+1, −*z*+1; (iii) −*x*+2, −*y*+1, −*z*.
